# State-dependent modulation of spiny projection neurons controls levodopa-induced dyskinesia in a mouse model of Parkinson’s disease

**DOI:** 10.1126/sciadv.adv8224

**Published:** 2025-12-03

**Authors:** Shenyu Zhai, Qiaoling Cui, David Wokosin, Linqing Sun, Tatiana Tkatch, Jill R. Crittenden, Ann M. Graybiel, D. James Surmeier

**Affiliations:** ^1^ Department of Neuroscience, Feinberg School of Medicine, Northwestern University, Chicago, IL 60611, USA.; ^2^ Aligning Science Across Parkinson’s (ASAP) Collaborative Research Network, Chevy Chase, MD 20815 USA.; ^3^ McGovern Institute for Brain Research and Department of Brain and Cognitive Sciences, MIT, Cambridge, MA 02139, USA.

## Abstract

In the later stages of Parkinson’s disease, patients often manifest levodopa-induced dyskinesia (LID), compromising their quality of life. The pathophysiology underlying LID is poorly understood, and treatment options are limited. To move toward filling this treatment gap, the intrinsic and synaptic changes in striatal spiny projection neurons (SPNs) triggered by the sustained elevation of dopamine (DA) during dyskinesia were characterized using electrophysiological, pharmacological, molecular, and behavioral approaches. Our studies revealed that the intrinsic excitability and functional corticostriatal connectivity of SPNs in dyskinetic mice oscillate between LID on- and off-states in a cell- and state-specific manner. Although triggered by levodopa, these oscillations in SPN properties depended on both dopaminergic and cholinergic signaling. Disrupting M1 muscarinic receptor signaling specifically in indirect pathway SPNs or deleting its downstream signaling partner CalDAG-GEFI blunted the levodopa-induced alterations in functional connectivity, enhanced the motoric benefits of levodopa, and attenuated LID severity.

## Teaser

Levodopa-induced remodeling of striatal circuitry drives dyskinesia in Parkinson’s disease.

## INTRODUCTION

In Parkinson’s disease (PD), the loss of the dopaminergic neurons in the substantia nigra pars compacta disrupts basal ganglia circuitry, leading to bradykinesia, rigidity, and tremor (*1*). In the early stages of PD, systemic levodopa administration boosts the production and release of dopamine (DA), effectively ameliorating motor symptoms. However, as the disease progresses, the levodopa dose needed to alleviate symptoms climbs, and the machinery regulating extracellular DA wanes in efficacy (*2*–*5*). As a result, DA signaling is dysregulated, rising markedly for hours after levodopa treatment and then falling to very low levels until the next dose is taken. This abnormal, slow oscillation in brain DA concentration triggers alterations in basal ganglia circuits that result in uncontrolled movements (i.e., dyskinesia) shortly after levodopa is taken. Several parts of the basal ganglia have been implicated in the emergence of levodopa-induced dyskinesia (LID), but there is a consensus that the striatum is a critical site of pathophysiology (*6*, *7*).

The principal neurons of the striatum are GABAergic spiny projection neurons (SPNs), which constitute ~90% of all striatal neurons. About half of SPNs—so-called direct pathway SPNs (dSPNs)—project directly to the output nuclei of the basal ganglia, promoting action selection. The other half—the indirect pathway SPNs (iSPNs)—project to the external segment of the globus pallidus and thus are indirectly connected to the output nuclei; activity in iSPNs is generally thought to suppress contextually inappropriate actions (*8*, *9*). Because of their differential expression of DA receptors, iSPNs and dSPNs are modulated by DA in opposite ways. In dSPNs, G_s/olf_-coupled D1 DA receptors (D1Rs) stimulate adenylyl cyclase (AC) and protein kinase A (PKA), increasing intrinsic excitability, enhancing glutamatergic synaptic transmission, and facilitating long-term synaptic potentiation (LTP). By contrast, in iSPNs, G_i_-coupled D2 DA receptors (D2Rs) inhibit AC and stimulate phospholipase C, decreasing intrinsic excitability, attenuating glutamatergic transmission, and promoting long-term synaptic depression (LTD) (*9*–*13*). In the healthy striatum, dopaminergic signaling is episodic, being linked to the initiation of actions and to the outcomes of actions (*14*). These transient signaling events are thought to be critical to connecting actions and their outcomes to modifications in axospinous synaptic strength that underlie the acquisition of habits and contextually appropriate goal-directed actions (*10*, *14*).

The impact of dopaminergic signaling on SPNs is normally modulated by a dynamic interaction with autonomously active, giant, cholinergic interneurons (ChIs). Acting through D2Rs, striatal DA release inhibits the autonomous spiking of ChIs and their release of acetylcholine (ACh) (*15*–*18*). The ACh released by ChIs acts on iSPNs and dSPNs in ways that counter those of DA. In dSPNs, which primarily express G_i_-coupled M4 muscarinic receptors (M4Rs), ACh signaling blunts the effects of D1R activation and promotes LTD induction (*19*–*21*), whereas, in iSPNs, which only express M1 muscarinic receptors (M1Rs), ACh signaling enhances somatic excitability, dendritic integration, and LTP induction (*22*–*24*). The M1R-mediated modulation of iSPN dendrites is dependent upon CalDAG-GEFI (CDGI), a striatum-enriched, Ca^2+^-activated guanine nucleotide exchange factor (*22*, *25*, *26*). Thus, the interaction between dopaminergic and cholinergic signaling modulates not only the moment-to-moment excitability of striatal ensembles coordinating purposeful movement but also the long-term changes in synaptic strength that guide future behavior.

In models of late-stage PD, where most of dopaminergic neurons innervating the striatum have been lost, there appears to be a sustained enhancement of ACh release by ChIs (*27*). This shift is attributable to an elevation in ChI intrinsic excitability, a strengthening of their excitatory glutamatergic input from the parafascicular nucleus, and a disinhibition of ACh release from ChI terminals (*28*–*31*). In animal models of PD, optogenetic or chemogenetic inhibition of ChIs alleviates motor deficits (*21*, *30*, *32*). Furthermore, optogenetic activation of ChIs in healthy mice induces a parkinsonian-like state (*33*). The hypothesis that elevated ACh release contributes to the hypokinetic features of PD is also supported by the clinical observation that muscarinic receptor antagonists are of symptomatic benefit (*34*). However, the involvement of ChIs in the dyskinesia induced by levodopa treatment is controversial (*35*–*37*). On one hand, boosting cholinergic signaling appears to attenuate LID. For example, enhancing M4R signaling in dSPNs attenuates LID severity (*20*, *38*). On the other hand, several studies suggest that ChI ablation or disruption of cholinergic signaling attenuates LID severity (*39*, *40*). One critical gap in these studies is an assessment of how cholinergic signaling and SPNs are changing between the period when mice are dyskinetic and striatal DA is high (on-state) and when mice are hypokinetic and striatal DA is low (off-state). It is highly likely that the activity of ChIs and cholinergic signaling in these two states are very different. Moreover, as DA and ACh normally work in concert to control long-term changes in the functional connectomes of SPN underlying learning, it could be that the aberrant interaction between these neuromodulators induced by high doses of levodopa leads to pathological “learning” and alterations in circuitry that carry over from one state to the next.

To help fill this fundamental gap in our understanding, a combination of electrophysiological, imaging, genetic, pharmacological, and behavioral approaches was used in a mouse model of LID. These studies demonstrated that LID on- and off-states were associated with bidirectional, cell-type–specific changes in intrinsic excitability and synaptic connectivity. In addition, these studies demonstrated that ACh release by ChIs was elevated in parkinsonian mice and in the off-state of LID mice. Moreover, the release of ACh by ChIs continued to be negatively modulated by D2Rs in tissue from dyskinetic mice, arguing that striatal cholinergic signaling was inhibited in the on-state. The dysregulation of cholinergic signaling was critical to the state-dependent alterations in dendritic excitability and dendritic spine architecture in iSPNs. Blunting the impact of ChIs on iSPNs not only attenuated dyskinetic behaviors but also enhanced the motoric benefit of levodopa treatment.

## RESULTS

### Intrinsic excitability and synaptic connectivity of dSPNs increased in the on-state of LID

**Fig. 1.**
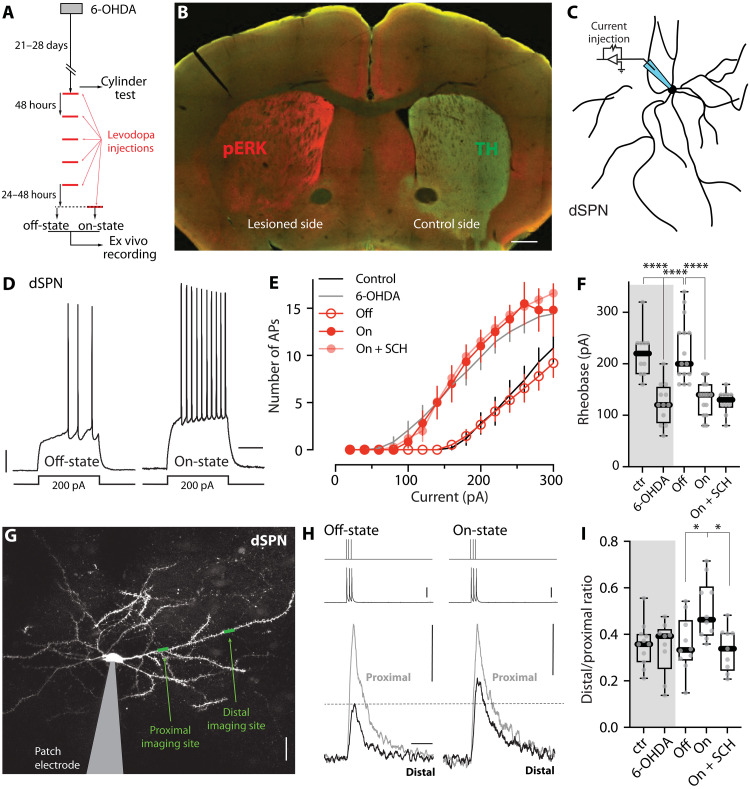
dSPN somatic and dendritic excitability changed between off- and on-states in LID mice. (**A**) Experimental timeline. LID mice were euthanized 24 to 48 hours or 30 min after the last levodopa injection for ex vivo assessment of the off- and on-state, respectively. (**B**) Confocal image of a coronal brain slice from a unilaterally 6-OHDA–lesioned, LID on-state mouse immunostained with anti-pERK (red) and anti–tyrosine hydroxylase (TH; green) antibodies. Scale bar, 0.5 mm. (**C**) Schematic illustrating the somatic excitability assay in dSPNs. (**D**) Sample voltage recordings from dSPNs in the off- and on-state of LID in response to a 200-pA current injection. Scale bars, 10 mV and 200 ms. (**E**) Current-response curves showing that somatic excitability of dSPNs was state dependent (control: *n* = 10 cells from six mice; 6-OHDA: *n* = 12 cells from 5 mice; off-state: *n* = 15 cells from 8 mice; on-state: *n* = 15 cells from 10 mice; on-state + SCH: *n* = 10 cells from 5 mice). (**F**) Box plot summary of rheobase in dSPNs. ****P < 0.0001; Mann-Whitney test. ctr, control. (**G**) Two-photon laser scanning microscopy (2PLSM) image of a patched dSPN with imaging sites on proximal and distal dendrites indicated. Scale bar, 20 μm. (**H**) Current injections of 2 nA (top) are aligned with evoked somatic spikes (middle) and fluorescence transients recorded from proximal and distal dendrites (bottom). Scale bars, 25 mV, 0.1 ΔG/R_0_, and 0.5 s. (**I**) Box plot summary of dendritic excitability index [the distal-to-proximal ratio of area under the curve (AUC) of ΔG/R_0_] of dSPNs (control: *n* = 11 cells from 6 mice; 6-OHDA: *n* = 9 cells from 5 mice; off-state: *n* = 10 cells from 7 mice; on-state: *n* = 10 cells from 5 mice; on-state + SCH: *n* = 9 cells from 5 mice). *P < 0.05; Mann-Whitney test.

Previous studies of how the induction of LID in rodents alters the intrinsic excitability and synaptic connectivity of SPNs have focused on the “off-state” (defined as 24 to 48 hours after the last administration of levodopa) (*41*–*44*). The implicit assumption of these studies is that while SPN properties change over the days during the induction of LID, they do not change in the relatively short period after termination of levodopa treatment. To test this hypothesis, we compared the properties of dSPNs shortly after levodopa treatment (on-state) and then again 24 to 48 hours after the last dose of levodopa (operationally defined as the off-state). *Drd1*-Tdtomato bacterial artificial chromosome (BAC) transgenic mice were rendered parkinsonian by unilateral medial forebrain bundle (MFB) injection of 6-hydroxydopamine (6-OHDA) (Fig. 1A). Three to 4 weeks later, the extent of DA denervation was assessed using a drug-free, forelimb-use asymmetry test (also called cylinder test). Mice with a near-complete lesion were given dyskinesiogenic doses of levodopa every other day for at least five sessions [6 mg/kg for the first two sessions and 12 mg/kg for later sessions, supplemented with benserazide (12 mg/kg)] (*4*, *20*, *41*). Mice were then euthanized either 30 min after the last levodopa dose (on-state) or 24 to 48 hours after the last dose (off-state). In the on-state, phosphorylated extracellular signal–regulated kinase (pERK) in the DA-depleted striatum was elevated (Fig. 1B), as previously reported (*45*, *46*).

To assess somatic excitability, we subjected visually identified dSPNs in brain slices to whole-cell patch clamp recording (Fig. 1C) (*20*, *41*, *47*). The response to current steps was monitored, and the relationship between step amplitude and evoked spiking (*F*-*I* relationship) was plotted (Fig. 1, D and E). Similar to what has been described in previous studies (*41*), somatic excitability of dSPNs was significantly increased following 6-OHDA lesioning and decreased by repetitive doses of levodopa when assessed in the off-state (both *P* < 0.0001) (Fig. 1, E and F). However, in brain slices taken shortly after levodopa treatment, dSPN somatic excitability was elevated. Specifically, in on-state dSPNs, the current needed to evoke a spike (rheobase) was significantly smaller than that in the off-state (*P* < 0.0001) (Fig. 1F), and the number of spikes evoked by suprathreshold current steps was greater across a range of intensities (Fig. 1E). This shift in somatic excitability was consistent with there being an elevation in extracellular DA shortly after levodopa treatment, resulting in activation of D1Rs on dSPNs (*48*–*53*). Furthermore, in agreement with recent work showing that the modulation of dSPN somatic excitability can persist for an extended period after termination of D1R stimulation (*54*), bath application of the D1R antagonist SCH 23390 (3 μM) did not reverse the leftward shift in the *F*-*I* relationship curve (Fig. 1E) or the reduction in rheobase (*P* = 0.589) (Fig. 1F).

Most of the dSPN surface area is dendritic, making this region a key site of dopaminergic modulation. To assess dSPN dendritic excitability in different LID states, we used a combination of patch clamp electrophysiology and two-photon laser scanning microscopy (2PLSM). Identified dSPNs in ex vivo brain slices were patch clamped, filled with the Ca^2+^-sensitive dye Fluo-4 and the Ca^2+^-insensitive dye Alexa Fluor 568 (to visualize dendrites), and then injected with brief current steps (three 2-nA injections, 2 ms each, at 50 Hz). These somatically delivered steps evoked action potentials (spikes) that backpropagate into SPN dendrites (*23*, *55*). To assess the spread of backpropagating action potentials (bAPs), we used 2PLSM to determine evoked changes in Fluo-4 fluorescence along dendrites produced by transient opening of voltage-dependent Ca^2+^ channels (*23*, *55*). The magnitudes of the Ca^2+^ signals at proximal (~40 μm from soma) and distal (~90 μm from soma) dendritic locations served as a surrogate estimate of the extent of dendritic depolarization produced by the bAPs (Fig. 1, G and H). To generate an estimate of bAP invasion that was independent of dye concentration, laser intensity, and other experimental variables, we normalized the bAP-evoked Ca^2+^ signal in a distal dendritic segment by the bAP-evoked Ca^2+^ signal in the proximal region of the same dendrite. This index of dendritic excitability was not significantly different in dSPNs from 6-OHDA–lesioned mice or LID mice in the off-state (control versus 6-OHDA, *P* = 0.824; 6-OHDA versus off-state, *P* = 0.905) (*23*). However, the dendritic excitability of dSPNs in the on-state was higher than in the LID off-state (*P* = 0.0115) (Fig. 1I), as expected from an elevation in D1R signaling (*52*, *53*). Unlike somatic excitability, the elevation in on-state dSPN dendritic excitability was readily reversed by bath application of the D1R antagonist SCH 23390 (*P* = 0.0133) (Fig. 1I).

**Fig. 2.**
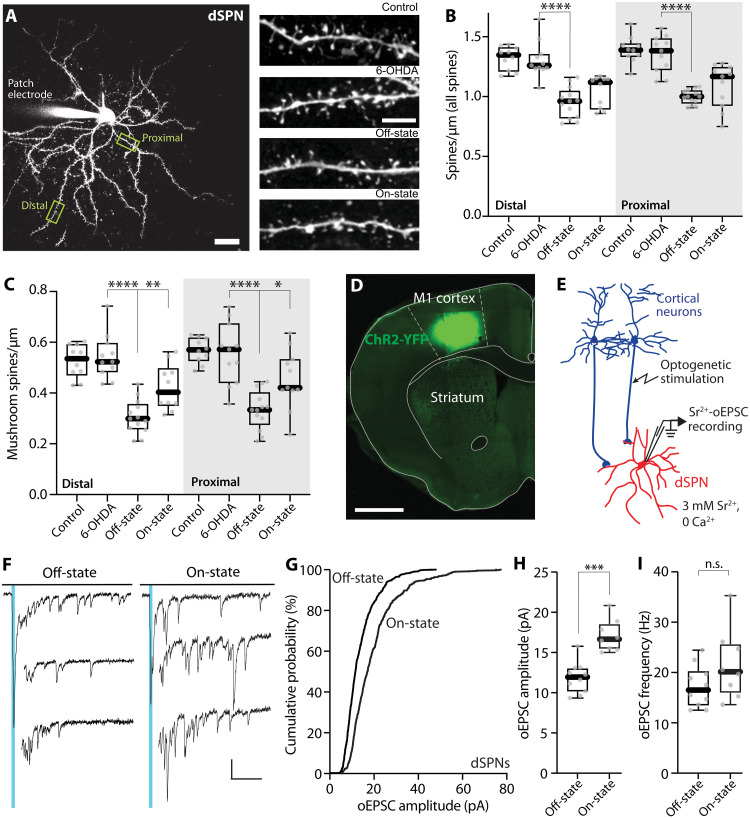
dSPN spine morphology and unitary synaptic strength changed between off- and on-states in LID mice. (**A**) Left: A low-magnification 2PLSM image of a patched dSPN with its dendritic tree visualized by Alexa Fluor dye and its proximal and distal locations used for spine density measurements delineated. Scale bar, 20 μm. Right: Sample 2PLSM images of dendritic segments of dSPNs. Scale bar, 5 μm. (**B** and **C**) Box plot summaries of density of all dendritic spines (B) and density of mushroom-type spines (C) in proximal and distal dendrites of dSPNs (control: *n* = 10 cells from six mice; 6-OHDA: *n* = 11 cells from five mice; off-state: *n* = 13 cells from eight mice; on-state: *n* = 10 to 11 cells from seven to eight mice). *P < 0.05, **P < 0.01, and ****P < 0.0001; Mann-Whitney test. (**D**) Confocal image of channelrhodopsin 2 (ChR2)–yellow fluorescent protein (YFP) expression in the motor cortex in a coronal section. Scale bar, 1 mm. (**E**) Schematic illustrating the experimental setup for Sr^2+^–optically evoked excitatory postsynaptic current (oEPSC) measurement. Whole-cell patch clamp recordings were made from dSPNs in acute brain slices of mice, and oEPSCs were evoked by brief blue light-emitting diode (LED) pulses. (**F**) Sample traces of Sr^2+^-oEPSC evoked by optogenetic stimulation of cortical afferents (indicated by blue vertical lines) in the presence of 3 mM Sr^2+^ and nominally 0 Ca^2+^ from dSPNs in LID off- and on-states. Scale bars, 20 pA and 100 ms. (**G**) Cumulative probability plot of Sr^2+^-oEPSC amplitudes in dSPNs from off- and on-state mice. (**H** and **I**) Box plots showing an increase in Sr^2+^-oEPSC amplitudes (H) in on-state dSPNs but not in Sr^2+^-oEPSC frequency (I) (off-state: *n* = 10 cells from four mice; on-state: *n* = 8 cells from four mice). ***P < 0.001 and n.s. (not significant); Mann-Whitney test.

In addition to modulating intrinsic excitability, dopaminergic signaling regulates the strength of glutamatergic synapses on dSPNs (*10*, *56*). Activation of D1Rs promotes the induction of LTP at axospinous, corticostriatal synapses, which can manifest itself as a structural enlargement of spines (*57*, *58*). To determine LID-associated structural changes in dendritic spines, we loaded dSPNs in ex vivo brain slices with Alexa Fluor 568 through the patch pipette and then optically dissected their dendritic morphology using 2PLSM (Fig. 2A) (*41*). Consistent with previous reports (*41*–*43*, *59*), dSPN spine density was not altered in either proximal or distal dendrites 3 to 4 weeks following unilateral, MFB 6-OHDA lesion (*P* = 0.654 and 0.756 for distal and proximal dendrites, respectively) (Fig. 2, A and B). However, in dSPNs taken from LID mice that were in the off-state, spine density in both dendritic regions was significantly reduced (*P* < 0.0001 in both proximal and distal dendrites) (Fig. 2, A and B). In dSPNs taken from on-state LID mice, overall spine density was not significantly different from that in the off-state (distal, *P* = 0.115; proximal, *P* = 0.106) (Fig. 2, A and B). However, in the on-state, enlarged, mushroom spines were more common in both distal and proximal dendrites (on-state versus off-state: distal, *P* = 0.0025; proximal, *P* = 0.0184) (Fig. 2C).

To determine whether these structural changes were accompanied by alterations in synaptic function, we used a combination of electrophysiological and optogenetic approaches (*30*, *60*, *61*). To selectively activate corticostriatal synapses, we injected an adeno-associated virus (AAV) carrying a channelrhodopsin 2 (ChR2) expression construct (AAV5-hSyn-hChR2(H134R)-eYFP) into the motor cortex ipsilateral to the 6-OHDA lesion (Fig. 2D). In brain slices from off- and on-state *Drd1*-Tdtomato mice, dSPNs were patched with a Cs^+^-containing intracellular solution and voltage clamped. To measure unitary synaptic strength, we replaced the extracellular Ca^2+^ with Sr^2+^ (3 mM), stimulated cortical fibers with blue light-emitting diode (LED) pulses, and recorded asynchronous, optically evoked excitatory postsynaptic currents (Sr^2+^-oEPSCs) (Fig. 2, E and F, E and F). Consistent with the elevation in mushroom spine density, the distribution of Sr^2+^-oEPSC amplitudes was shifted toward larger values, and the Sr^2+^-oEPSC amplitude was significantly larger in on-state dSPNs than in off-state dSPNs (*P* = 0.0003) (Fig. 2, F to H). Although the frequency of on-state Sr^2+^-oEPSCs trended toward higher values (Fig. 2I), as did spine density (Fig. 2, B and C), the differences between states were not statistically significant (*P* = 0.127). Together, these observations suggest that on-state dopaminergic signaling in dyskinetic mice leads to an enhancement in intrinsic excitability and a potentiation of corticostriatal synaptic function in dSPNs, both of which are consistent with what is known about how D1R activation modulates excitability and synaptic plasticity in dSPNs from healthy mice (*48*, *57*).

### Intrinsic excitability and synaptic connectivity of iSPNs decreased in the on-state of LID

**Fig. 3.**
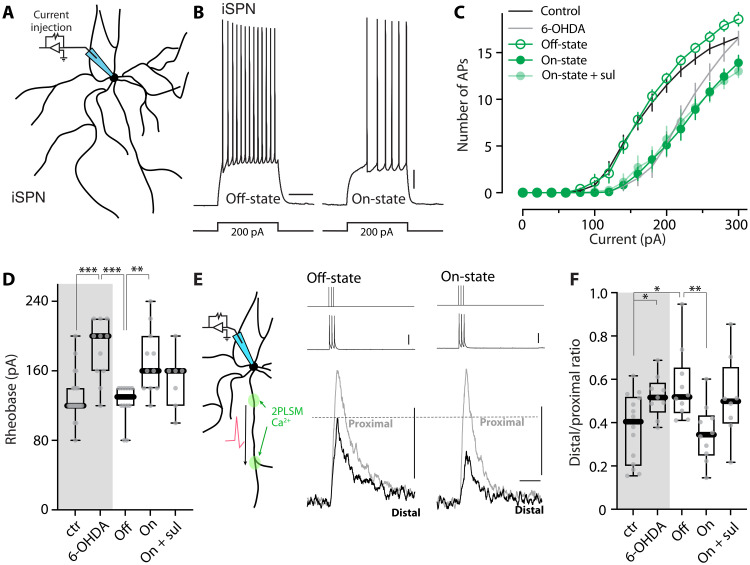
Somatic and dendritic excitability of iSPNs changed between off- and on-states in LID mice. (**A**) Schematic illustrating the somatic excitability assay in iSPNs. (**B**) Sample voltage recordings from off- and on-state iSPNs in response to a 200-pA current injection (500-ms duration). Scale bars, 10 mV and 200 ms. (**C**) Current-response curves showing the decrease in iSPN somatic excitability in the on-state compared to off-state (control: *n* = 21 cells from 10 mice; 6-OHDA: *n* = 11 cells from 6 mice; off-state: *n* = 12 cells from 5 mice; on-state: *n* = 11 cells from 8 mice; on-state + D2R antagonist: *n* = 7 cells from 4 mice). (**D**) Box plot summary of rheobase in iSPNs. **P < 0.01 and ***P < 0.001; Mann-Whitney test. (**E**) Left: Schematic illustrating the dendritic excitability assay in iSPNs. Right: Fluo-4 fluorescence transients (bottom) recorded from iSPN proximal and distal dendrites are aligned with current injections of 2 nA (top) and corresponding somatic spikes (middle). Scale bars, 25 mV, 0.1 ΔG/R_0_, and 0.5 s. (**F**) Box plot summary of dendritic excitability index (the distal-to-proximal ratio of AUC of ΔG/R_0_) in iSPNs from control, 6-OHDA–lesioned, off-state, on-state, or on-state mice with bath application of sulpiride (control: *n* = 14 cells with eight mice; 6-OHDA: *n* = 9 cells from six mice; off-state: *n* = 10 cells from five mice; on-state: *n* = 10 cells from five mice; on-state + sulpiride: *n* = 8 cells from six mice). *P < 0.05 and **P < 0.01; Mann-Whitney test. sul, sulpiride.

Although it is clear that dSPNs are critical to the emergence of LID, iSPNs are also pivotal to this condition (*62*, *63*). Clear changes in the properties of iSPNs following LID induction have been reported, but previous assessments have been limited to the off-state, leaving unexplored how they are modulated in the on-state (*41*–*44*). To fill this gap, we studied iSPNs using *Drd2*-enhanced green fluorescent protein (eGFP) mice following unilateral 6-OHDA lesioning and levodopa treatments. Somatic excitability was assessed using the same strategy as described above (Fig. 3, A and B). Consistent with our previous report (*41*), somatic excitability of iSPNs was lowered by DA depletion and restored by repetitive doses of levodopa when assessed in the off-state (both *P* = 0.002) (Fig. 3, C and D). On-state iSPNs were found to be less excitable than those in the off-state (Fig. 3, B to D): Rheobase was elevated compared to the off-state (*P* = 0.0013) (Fig. 3D), and the *F*-*I* relationship curve shifted to the right (Fig. 3C). Acute antagonism of D2Rs with sulpiride (10 μM) in slices taken from on-state mice did not alter somatic excitability (rheobase; *P* = 0.287), arguing that the hypoexcitability was not being maintained by DA (Fig. 3, C and D).

To complement the assessment of somatic excitability, we also examined the dendritic excitability of iSPNs using the combination of patch clamp electrophysiology and 2PLSM, as described above. Consistent with our previous report (*23*), the index of dendritic excitability in iSPNs increased after DA depletion and stayed elevated after LID induction and termination of levodopa treatment (i.e., off-state) (control versus 6-OHDA lesion, *P* = 0.0327; control versus off-state, *P* = 0.0220). However, in the on-state, iSPN dendritic excitability fell (*P* = 0.0029) (Fig. 3, E and F). Acute blockade of dopaminergic signaling did not significantly alter dendritic excitability in on-state iSPNs (*P* = 0.0545) (Fig. 3F), suggesting that some other factor was involved.

**Fig. 4.**
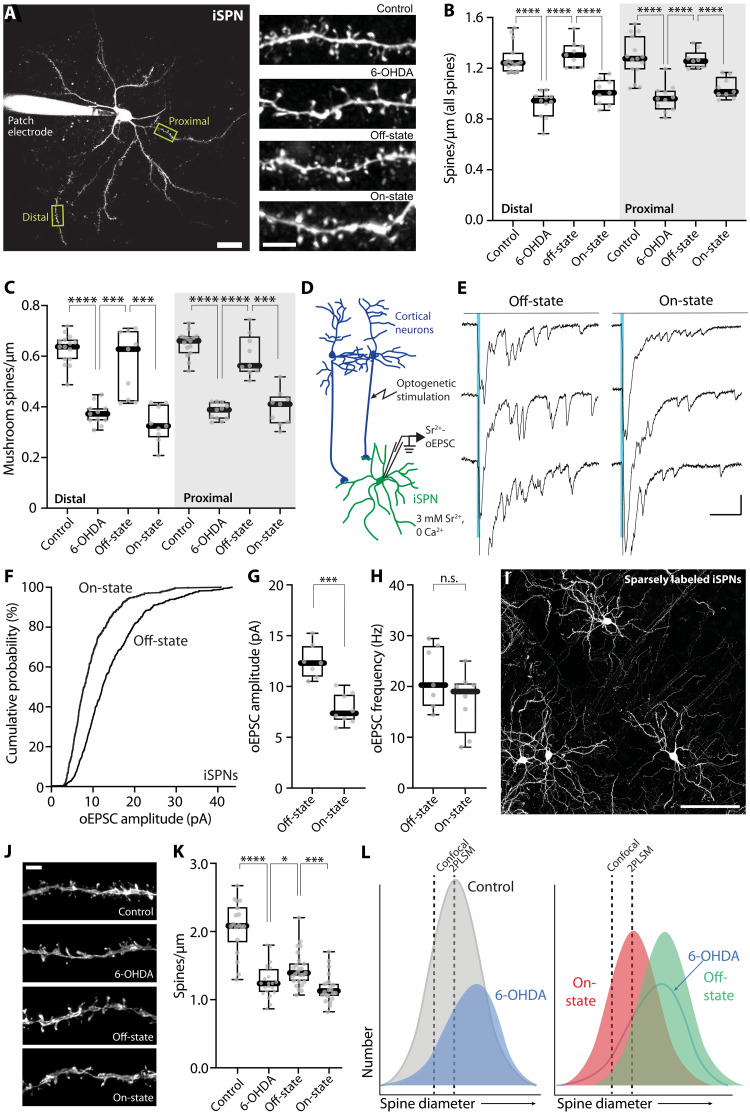
Spine morphology and unitary synaptic strength of iSPNs changed between off- and on-states in LID mice. (**A**) Left: 2PLSM image of a patched iSPN with its proximal and distal dendritic segments delineated. Scale bar, 20 μm. Right: 2PLSM images of iSPN dendrites. Scale bar, 5 μm. (**B** and **C**) Box plot summaries of total (B) and mushroom (C) spine densities in proximal and distal dendrites of iSPNs (control: *n* = 15 cells from nine mice; 6-OHDA: *n* = 11 cells from six mice; off-state: *n* = 9 cells from five mice; on-state: *n* = 9 cells from five mice). (**D**) Schematic illustrating the experimental setup for Sr^2+^-oEPSC measurement. (**E**) Representative recordings of Sr^2+^-oEPSC evoked by optogenetic stimulation of cortical afferents (indicated by blue vertical lines) from iSPNs of off- and on-state mice. Scale bars, 20 pA and 100 ms. (**F**) Cumulative probability plot of Sr^2+^-oEPSC amplitudes. (**G** and **H**) Box plot summaries of Sr^2+^-oEPSC amplitudes (G) and frequencies (H) in iSPNs (off-state: *n* = 7 cells from four mice; on-state: *n* = 8 cells from four mice). (**I**) Confocal image of sparsely labeled iSPNs in a control mouse. Scale bar, 100 μm. (**J**) Representative high-resolution confocal images showing proximal dendrites of sparsely labeled iSPNs. Scale bar, 3 μm. (**K**) Box plot summary of spine density in proximal dendrites of iSPNs imaged by high-resolution confocal microscopy (control: *n* = 18 dendrites from four mice; 6-OHDA: *n* = 22 dendrites from three mice; off-state: *n* = 25 dendrites from four mice; on-state: *n* = 22 dendrites from four mice). (**L**) Schematic showing different distributions of iSPN spine size in control, 6-OHDA–lesioned, off-, and on-state mice, as well as the detection thresholds of 2PLSM and confocal microscopy. *P < 0.05, ***P < 0.001, and ****P < 0.0001, and n.s. (not significant); Mann-Whitney test.

As with D1Rs in dSPNs, D2R signaling in iSPNs modulates glutamatergic synaptic plasticity (*9*, *10*, *56*). Activation of D2Rs promotes the induction of LTD at axospinous, corticostriatal synapses (*57*, *64*), which in principle should lead to a reduction in spine size (*65*). In addition, homeostatic mechanisms have been posited to regulate iSPN spine density (*24*, *66*). As a first step toward determining whether there were alterations in spine density or size in the on-state, iSPNs in ex vivo brain slices from *Drd2*-eGFP mice were examined after 6-OHDA lesioning, LID induction, and then termination of levodopa treatment (off-state). In agreement with previous work (*24*, *41*, *43*, *66*, *67*), spine density in both proximal and distal dendrites was significantly reduced 3 to 4 weeks after a 6-OHDA lesion (*P* < 0.0001 in both proximal and distal dendritic locations) (Fig. 4, A and B). After LID induction, off-state iSPN spine density returned to a range that was indistinguishable from that of iSPNs from mice without 6-OHDA lesions (Fig. 4, A and B), as previously reported (*41*, *67*). Unexpectedly, in the on-state, the apparent spine density measured with 2PLSM fell significantly in both proximal and distal iSPN dendrites (on-state versus off-state: *P* < 0.0001 for both dendritic locations) (Fig. 4, A and B), suggesting that axospinous synapses were being added in the off-state and removed in the on-state. Similar changes were observed in the density of mushroom spines (on-state versus off state: distal, *P* = 0.0006; proximal, *P* = 0.0002) (Fig. 4C).

If there were oscillations in the number of axospinous synapses between on- and off-states, then there should be a corresponding change in the frequency of asynchronous oEPSCs generated by activation of the corticostriatal pathway when extracellular Ca^2+^ was replaced with Sr^2+^. To test this hypothesis, we induced M1 cortical pyramidal neurons to express ChR2 as described above, and asynchronous, Sr^2+^-oEPSCs were recorded in iSPNs in ex vivo brain slices taken from mice in either the LID on- or off-states (Fig. 4, D and E). Unexpectedly, the amplitude, but not the frequency, of Sr^2+^-oEPSCs was significantly smaller in on-state iSPNs than in off-state iSPNs (amplitude: *P* = 0.0003; frequency: *P* = 0.23) (Fig. 4, F to H). These observations are consistent with the inference that what is changing in iSPNs between on- and off-states is the strength, but not the number, of corticostriatal axospinous synapses.

How can these seemingly disparate experimental results be reconciled? An obvious caveat to the 2PLSM counts is that thin spines can be missed. The density of SPN dendritic spines observed with high-voltage electron microscopy (HVEM) is considerably greater than that estimated from three-dimensional 2PLSM reconstructions (*68*, *69*). Although HVEM was beyond our experimental reach, high-resolution confocal microscopy with fixed brain tissue (see Materials and Methods) does offer an alternative approach. To this end, *Adora2*-Cre mice were injected with a diluted AAV carrying a Cre-dependent eGFP expression construct (AAV9-pCAG-flex-EGFP-WPRE). This led to sparse labeling of iSPNs, allowing visualization of individual dendrites (Fig. 4, I and J). Three-dimensional confocal reconstruction of iSPNs yielded spine density estimates in proximal dendrites that were notably higher than those estimated from 2PLSM optical sectioning of live tissue (Fig. 4K) (confocal microscopy: median 2.084 versus 2PLSM: median 1.243; spines per micrometers). After 6-OHDA lesioning, iSPN spine density estimated from confocal imaging decreased (control versus 6-OHDA: *P* < 0.0001), consistent with the 2PLSM estimates and previous reports (*24*, *41*, *43*, *66*, *67*). However, in contrast to the conclusions drawn from 2PLSM, confocal spine density estimates did not return to control values in the off-state after LID induction, although they did increase modestly from the 6-OHDA–lesioned state (*P* = 0.0136) (Fig. 4, J and K). As with 2PLSM, confocal estimates of iSPN spine density fell in the on-state (*P* = 0.0001) (Fig. 4, J and K) but only modestly.

What these observations suggest is that the apparent fluctuations in iSPN spine density following LID induction largely reflect alterations in the size—and detectability—but not the number of axospinous synapses. To illustrate how this might occur, we hypothesized the distribution of spine diameters to be Gaussian (Fig. 4L). Given the limitations of light microscopy, there is a threshold diameter for spine detection; this threshold should be greater for 2PLSM, which used a lens with relatively smaller numerical aperture (NA) than that used for the confocal analysis (0.9 NA 60× lens for 2PLSM and 1.49 NA 60× lens for confocal microscopy); hypothetical thresholds are drawn on the distributions to show how in principle they should change spine density estimates (Fig. 4L). After DA depletion, the distribution of spine diameters should change in two ways. First, the total number of synapses should drop by about 30% to reflect frank spine pruning. Second, the distribution of diameters should shift slightly toward larger values to account for the aberrant potentiation of axospinous synapses accompanying loss of iSPN D2R signaling (Fig. 4L) (*41*). To illustrate the alterations between on- and off-states, we simply shifted the distribution of spine diameters to the right (larger) to reflect synaptic potentiation in the off-state and to the left (smaller) to reflect synaptic depression in the on-state (Fig. 4L). This simple model accounts for the apparent alterations in spine density estimated with 2PLSM, as well as the Sr^2+^-oEPSC data.

### ACh Release was elevated in PD and LID off-state and inhibited by D2R signaling

The data presented to this point argue that dyskinesiogenic fluctuations in dopaminergic signaling between the on- and off-states drive cell-specific changes in the intrinsic excitability and functional connectivity of iSPNs and dSPNs. Fluctuations in brain DA trigger these changes, but it is unclear whether, at the level of individual SPNs, DA is acting alone. That is, are there other alterations in the striatal circuitry induced by levodopa treatment that contribute to the observed changes in the functional properties of SPNs? One key target of intrastriatal dopaminergic signaling is the ChI. Autonomously active ChIs are robustly inhibited by DA acting on D2Rs (*15*–*18*, *70*). Moreover, ACh potently modulates the intrinsic excitability and synaptic plasticity of iSPNs and dSPNs through mechanisms that complement those of DA (*71*, *72*). However, the role of ChIs in the parkinsonian state and LID is controversial [reviewed in (*27*, *35*–*37*)].

**Fig. 5.**
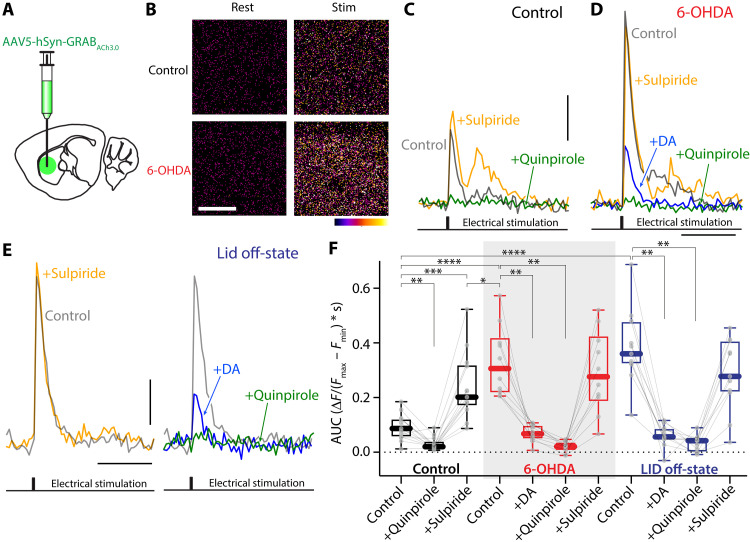
ACh release was increased in the striata of PD and LID mice but strongly suppressed by DA. (**A**) Diagram showing injection of GRAB_ACh3.0_-expressing AAV into the DLS of mice. (**B**) Pseudocolor images of GRAB_ACh3.0_ signal (Δ*F*/*F*_0_) in resting state (“rest”) and immediately following stimulation (“stim”) in the DLS of unlesioned (“control”) or 6-OHDA–lesioned (“6-OHDA”) mice. Scale bar, 20 μm. (**C** to **E**) Representative traces of the fluorescence response of GRAB_ACh3.0_ signals in the DLS of control (C), 6-OHDA–lesioned (D), and LID off-state mice (E) evoked by an intrastriatal electrical stimulation before and after bath application of D2R agonist(s) (quinpirole or DA) or D2R antagonist sulpiride. Scale bars, 1 s and 0.2 Δ*F*/*F*_0_. (**F**) Box plot summary of GRAB_ACh3.0_ signal [AUC of Δ*F*/(*F*_max_ – *F*_min_)] in unlesioned control, 6-OHDA–lesioned, and LID off-state mice. In 6-OHDA–lesioned and off-state mice, ACh release was significantly elevated. Bath application of DA (50 nM), mimicking the high DA condition in LID on-state, strongly suppressed ACh release [unlesioned control: *n* = 13 regions of interest (ROIs) from three mice; 6-OHDA: *n* = 10 ROIs from three mice; off-state: *n* = 11 ROIs from five mice]. *P < 0.05, **P < 0.01, ***P < 0.001, and ****P < 0.0001; Mann-Whitney (unpaired) and Wilcoxon (paired) tests.

To directly assess ACh release by ChIs in the parkinsonian and LID states, we used the genetically encoded ACh sensor GRAB_ACh3.0_ in conjunction with 2PLSM (*73*). About 3 weeks after stereotaxic injection into the dorsolateral striatum (DLS) of an AAV carrying a GRAB_ACh3.0_ expression construct, ex vivo brain slices were prepared, and sensor fluorescence was monitored in response to local electrical stimulation (Fig. 5, A and B). Robust ACh signals were evoked by either single pulses (Fig. 5) or short pulse bursts (fig. S1). In the healthy DLS, the ACh signal rose rapidly and decayed back to baseline within 1 to 2 s (Fig. 5C). Bath application of the D2R antagonist sulpiride (10 μM) increased the evoked ACh signal (*P* = 0.0005) (Fig. 5, C and F, and fig. S1), indicating that there was a basal dopaminergic tone. As expected, bath application of a D2R agonist quinpirole (10 μM) suppressed the ACh signal (*P* = 0.0034) (Fig. 5, C and F).

In ex vivo slices from 6-OHDA–lesioned mice, the GRAB_ACh3.0_ signal was markedly elevated (unlesioned versus 6-OHDA: *P* < 0.0001) (Fig. 5, B, D, and F, and fig. S1), suggesting that ACh release was disinhibited by DA depletion. Consistent with this scenario, bath application of sulpiride (10 μM) had no effect (*P* = 0.5566), whereas D2R agonists (50 nM DA or 10 μM quinpirole) strongly inhibited ACh release in 6-OHDA mice (DA: *P* = 0.002; quinpirole: *P* = 0.002) (Fig. 5, D and F, and fig. S1). In slices from LID off-state mice, ACh release was significantly greater than that in the unlesioned striatum (*P* < 0.0001) but similar to that following a 6-OHDA lesion (*P* = 0.4679). Bath application of DA (50 nM), at a concentration comparable to that detected in the striatum in vivo during on-state (*74*–*76*), strongly suppressed ACh release (*P* = 0.001) (Fig. 5, E and F, and fig. S1). These data suggest that ACh release falls in the on-state, as intrastriatal DA levels rise and then rebound in the off-state when DA levels drop.

### M1 muscarinic receptor–CDGI signaling contributed to iSPN plasticity and LID induction

How might the dysregulation of ACh release have contributed to the changes in SPN properties found in LID on- and off-states? The intrinsic excitability and synaptic function of both iSPNs and dSPNs are potently modulated by muscarinic ACh receptors (mAChRs). Previous work has implicated an imbalance in the activity of dSPN D1Rs and M4Rs during the on-state in LID induction (*20*, *38*), but how alterations in iSPN properties were influenced by disrupted cholinergic signaling is less clear cut.

**Fig. 6.**
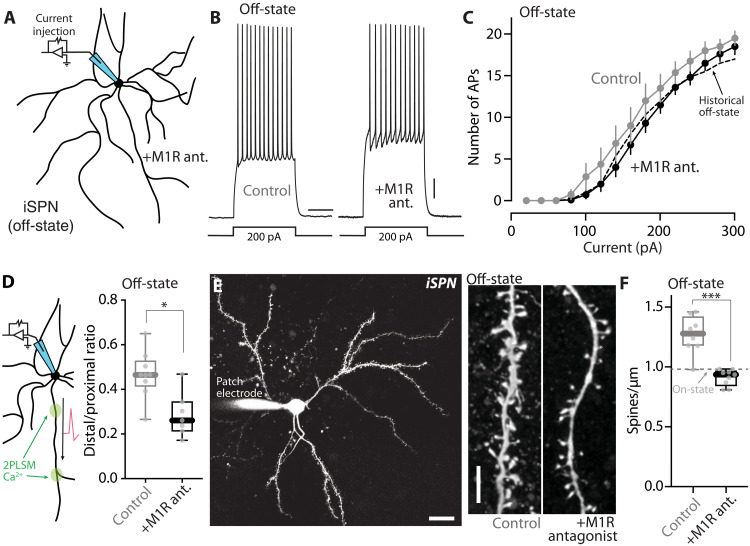
M1R mediated the dendritic, but not the somatic, alterations in iSPNs in the off-state in LID mice. (**A**) Schematic illustrating the somatic excitability assay in iSPNs from LID off-state mice in which M1R signaling was inhibited throughout the off-state. (**B**) Sample voltage changes evoked by 200-pA current injections (500-ms duration) in iSPNs from off-state mice that received M1R antagonist (“+M1R ant.”) or vehicle control throughout the off-state. Scale bars, 10 mV and 200 ms. (**C**) Current-response curves showing that iSPN somatic excitability in off-state was resistant to M1R antagonists (off-state control: *n* = 8 cells from four mice; off-state with M1R antagonists: *n* = 10 cells from five mice). Historical off-state data from Fig. 3C were overlaid as a dashed line. (**D**) Left: Schematic illustrating the dendritic excitability assay in iSPNs. Right: Summary of dendritic excitability index in iSPNs from off-state mice treated with M1R antagonist or vehicle control (off-state control: *n* = 9 cells from five mice; off-state + M1R antagonists: *n* = 7 cells from four mice). *P < 0.05; Mann-Whitney test. (**E**) Left: Low-magnification image showing an iSPN from an off-state mouse treated with M1R antagonists, patched, and filled with Alexa Fluor dye. Scale bar, 20 μm. Right: Sample 2PLSM images of dendritic segments of iSPNs from off-state mice treated with vehicle (control) or M1R antagonists (“+M1R antagonist”). Scale bar, 5 μm. (**F**) Box plot summary of dendritic spine density in off-state iSPNs treated with vehicle or M1R antagonists (control: *n* = 8 cells from four mice; +M1R antagonist: *n* = 9 cells from five mice). ***P < 0.001; Mann Whitney test. Historical on-state data from Fig. 4B were shown by a dashed line.

In normal mice, activation of iSPN M1Rs enhances both somatic and dendritic excitability (*22*–*24*). Genetic deletion of the gene coding for CDGI, which couples M1Rs to intracellular signaling pathways, blunts the M1R-induced modulation of iSPNs dendritic excitability and prevents the induction of LTP at corticostriatal glutamatergic synapses (*22*). To assess the role of M1Rs in LID-induced adaptations in iSPNs, we gave dyskinetic *Drd2*-eGFP mice intraperitoneal injections of the M1R antagonist trihexyphenidyl hydrochloride (THP; 3 mg/kg) or saline right before the off-state started (~2 hours after levodopa administration); THP is known to have good brain bioavailability and favorable pharmacokinetics after peripheral administration (*77*). Mice were euthanized 16 hours later, ex vivo brain slices were prepared, and iSPNs were patch clamped (Fig. 6A). The slices from mice given THP were continuously incubated with the M1R-selective antagonist VU 0255035 (5 μM) throughout the experiment. Somewhat unexpectedly, M1R antagonism did not have a strong impact on the elevation in iSPN somatic excitability in the off-state (Fig. 6, B and C). However, M1R antagonism did prevent the off-state elevation in dendritic excitability (*P* = 0.012) (Fig. 6D) and the apparent elevation in spine density (*P* = 0.0003) (Fig. 6, E and F).

**Fig. 7.**
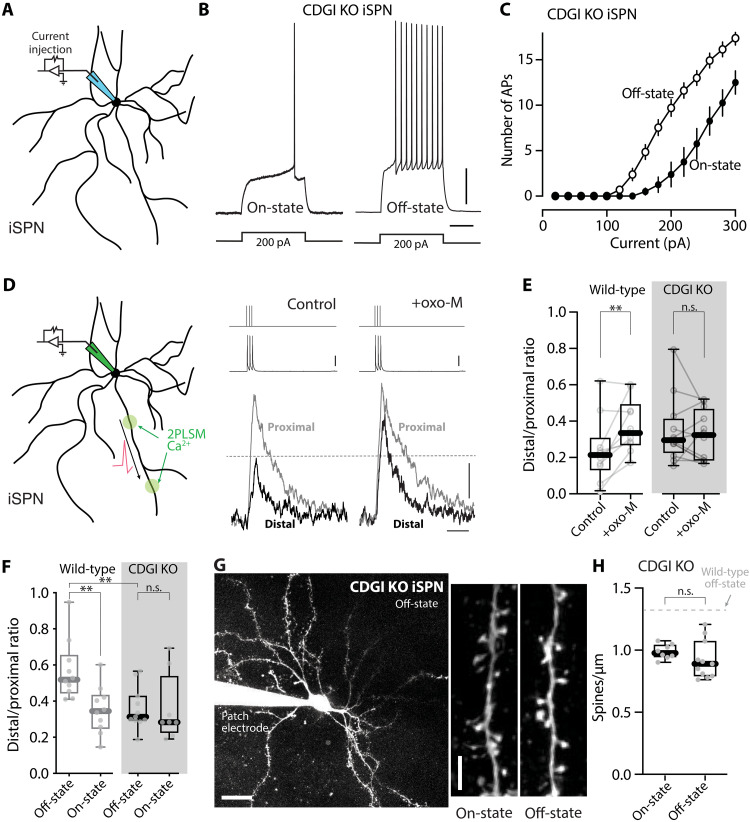
CDGI signaling pathway mediated the dendritic changes in off-state iSPNs. (**A**) Schematic of somatic excitability assay. (**B**) Sample voltage recordings from on- and off-state iSPNs from CDGI KO mice. Scale bars, 10 mV and 200 ms. (**C**) Current-response curves of on- and off-state iSPNs from CDGI KO (on-state: *n* = 8 cells from four mice; off-state: *n* = 13 cells from five mice). (**D**) Left: Schematic of dendritic excitability assay. Right: Fluo-4 signals from iSPN proximal and distal dendrites before and after oxo-M application in slices from wild-type mice. Current injections (2 nA) and somatic spikes were shown on top. Scale bars, 25 mV, 50% Δ*F*/*F*_0_, and 0.5 s. (**E**) Box plot summary showing the effect of oxo-M on iSPN dendritic excitability in wild-type and CDGI KO mice (*n* = 10 to 12 dendrites from three mice per genotype). **P < 0.01 and n.s. (not significant); Wilcoxon test. (**F**) Box plot summary of dendritic excitability index of iSPNs from wild-type or CDGI KO mice in off- or on-state (wild-type data were from Fig. 3F; KO off-state: *n* = 11 cells from five mice; KO on-state: *n* = 8 cells from four mice). **P < 0.01 and n.s. (not significant); Mann-Whitney test. (**G**) Left: Image of a patched iSPN from an off-state CDGI KO mouse. Scale bar, 20 μm. Right: Images of iSPN dendrites from CDGI KO mice. Scale bar, 5 μm. (**H**) Box plot summary of iSPN spine density in on- and off-state CDGI KO mice (on-state: *n* = 8 cells from four mice; off-state: *n* = 11 cells from six mice). n.s. (not significant); Mann-Whitney test.

These observations suggest that M1R signaling contributes to the LID-induced adaptations in iSPNs, particularly those in dendrites. The obvious limitations of these experiments are that THP is not selective for M1Rs (*78*) and that M1Rs are broadly distributed in the brain—raising the possibility that the effects of THP are not dependent upon iSPN M1Rs. As a first step toward addressing these limitations, CDGI knockout (KO) mice were studied; CDGI is largely restricted to the striatum and is a key mediator of dendritic M1R signaling in iSPNs (*22*, *25*). CDGI KO mice were subjected to the same LID induction protocol as described above, and then brain slices were prepared for study. In agreement with its dendritic role (*22*), deletion of CDGI did not blunt the oscillation in iSPN somatic excitability between LID on- and off-states (Fig. 7, A to C). To assess dendritic excitability, we examined bAP propagation. In ex vivo brain slices from naïve wild-type mice, bath application of the mAChR agonist oxotremorine M (oxo-M, 10 μM) robustly increased the invasion of bAPs into the dendrites of iSPNs (*P* = 0.0098) (Fig. 7, D and E), in agreement with previous work (*23*). However, in ex vivo slices from naïve mice lacking CDGI, the M1R agonist oxo-M had no effect on dendritic invasion of bAPs (*P* = 0.910) (Fig. 7E). Consistent with this observation, the enhancement of bAP dendritic invasion in wild-type iSPNs in the LID off-state was not seen in off-state CDGI KO iSPNs (off-state versus on-state: wild-type, *P* = 0.0029; CDGI KO, *P* = 0.395) (Fig. 7F). Furthermore, the apparent increase in iSPN spine density observed in wild-type mice in the off-state (Fig. 4B) was not seen in iSPNs from CDGI KO mice (off-state versus on-state: CDGI KO, *P* = 0.613) (Fig. 7, G and H). Together, these observations suggest that dendritic M1R/CDGI signaling in iSPNs plays a key role in the structural and functional adaptations accompanying the off-state in LID mice.

**Fig. 8.**
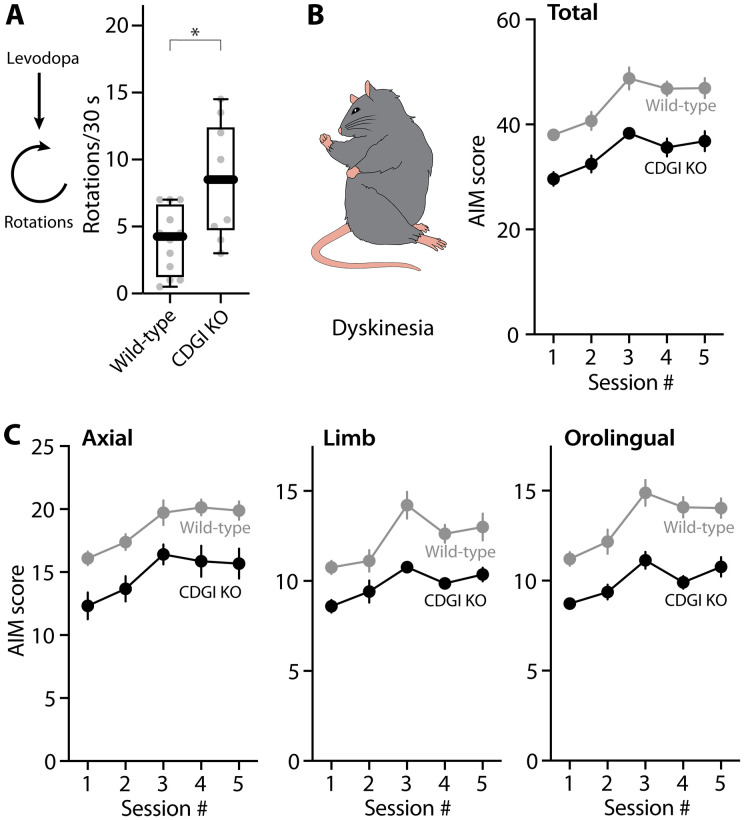
Genetic deletion of CDGI enhanced the motoric effect of levodopa and attenuated dyskinetic behaviors. (**A**) Box plot summary of the number of contralateral rotations (in 30 s) recorded 40 min after the fifth levodopa administration in wild-type or CDGI KO mice (wild-type: *n* = 12 animals; CDGI KO: *n* = 10 animals). *P < 0.05; Mann-Whitney test. (**B** and **C**) Plots of total (B), axial, limb, and orolingual (C) AIM scores as a function of sessions in wild-type and CDGI KO mice (wild-type: *n* = 12 mice; CDGI KO: *n* = 11 mice; data are mean ± SEM). Total AIM score: time *P* < 0.001, *F*(4,84) = 17.19; group *P* < 0.001, *F*(1,21) = 41.87. Axial score: time *P* < 0.001, *F*(2.429,51) = 11.32; group *P* = 0.002, *F*(1,21) = 19.91. Limb score: time *P* < 0.001, *F*(3.019,63.40) = 13.00; group *P* < 0.001, *F*(1,21) = 25.65. Orolingual score: time *P* < 0.001, *F*(3.192,67.04) = 13.84; group *P* < 0.001, *F*(1,21) = 56.29. Repeated-measures two-way analysis of variance (ANOVA) and post hoc Bonferroni test were used.

To determine whether these CDGI-dependent adaptations were linked to behavior, we compared the dyskinesia induced in wild-type and KO mice using the protocol described above. CDGI KO and wild-type controls were subjected to a unilateral 6-OHDA lesion and, about a month later, evaluated for the extent of the lesion using the cylinder test. Mice with a near-complete lesion were then injected with dyskinesiogenic doses of levodopa every other day, and their abnormal involuntary movements (AIMs) were rated using an established rating scale (*4*, *20*, *41*). Routinely, the prokinetic effects of levodopa were assessed by monitoring the number of contralateral rotations in a 30-s period during peak-dose dyskinesia (*79*–*82*). The number of contralateral rotations induced by levodopa was significantly greater in CDGI KO mice than in wild types (*P* = 0.0092 at the fifth dose) (Fig. 8A, fig. S2A, and movie S1). Axial, limb, and orolingual AIMs, as well as total AIMs, were less severe in CDGI KO mice than wild-type controls (Fig. 8, B and C, and movie S1). These results suggest that CDGI-dependent M1R signaling in iSPN dendrites makes an important contribution to the network pathophysiology underlying LID.

**Fig. 9.**
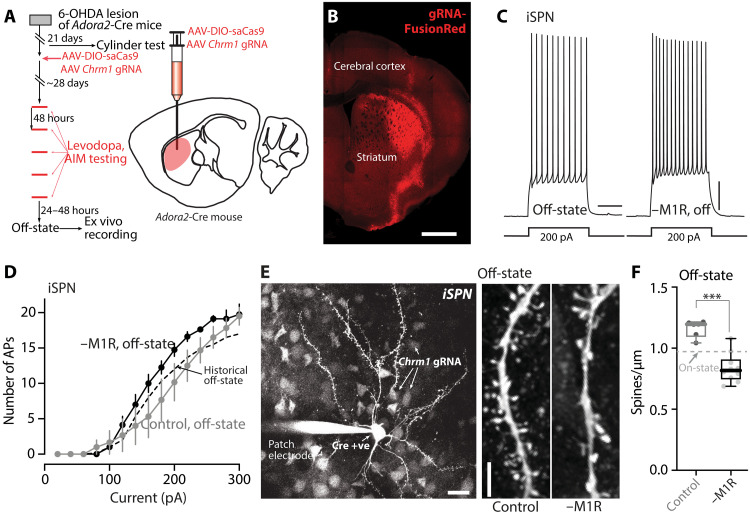
Deletion of M1R from iSPNs prevented dendritic changes in the off-state. (**A**) Left: Experimental timeline for 6-OHDA lesioning, M1R CRISPR expression, AIM testing, and ex vivo recordings. Right: Schematic illustrating injection of M1R CRISPR into the DLS of *Adora2*-Cre mice. (**B**) Confocal image showing expression of gRNA-FR in the DLS of a coronal section. Scale bar, 1 mm. (**C**) Sample somatic voltage changes in response to 200-pA current injections in iSPNs from off-state mice without or with M1R deletion from iSPNs. Scale bars, 10 mV and 200 ms. (**D**) Current-response curves showing that the increase in iSPN somatic excitability in the off-state was not prevented by M1R deletion (control: *n* = 6 cells from three mice; M1R deletion: *n* = 8 cells from four mice). Historical off-state data from Fig. 3C were shown by a dashed line. (**E**) Left: Low-magnification image showing a patched iSPN visualized by Alexa Fluor dye and expression of gRNA-FR in nearby cells in a nonspecific manner. Cell-type–specific deletion was achieved by Cre-dependent expression of saCas9 in *Adora2*-Cre mice. Scale bar, 20 μm. Right: 2PLSM images of dendritic segments of iSPNs from off-state mice without or with M1R deletion. Scale bar, 5 μm. (**F**) Box plot summary of dendritic spine density of iSPNs without or with M1R deletion from off-state mice (control: *n* = 6 cells from three mice; M1R deletion: *n* = 10 cells from four mice). ***P < 0.001; Mann-Whitney test. Historical on-state data from Fig. 4B were indicated by a dashed line.

Although these results implicate iSPNs in LID, it is possible that the loss of CDGI in other cell types (e.g., dSPNs) was a factor in the attenuation of LID. To address this caveat, we used a CRISPR-saCas9 approach to disrupt the expression of M1R specifically in iSPNs. In short, a mixture of two AAVs was injected into six sites across the ipsilateral DLS of unilaterally 6-OHDA–lesioned *Adora2*-Cre mice that had passed the cylinder test (Fig. 9A); one of the AAVs carried a Cre-dependent saCas9 expression construct, and the other carried *Chrm1*-targeting guide RNAs (gRNAs) with a FusionRed (FR) reporter construct. Control mice were injected with only the gRNA-FR vector (Fig. 9A). In a subset of experiments in which ex vivo analysis was to be performed, a third AAV carrying a Cre-dependent enhanced yellow fluorescent protein (eYFP) reporter (AAV9-EF1a-DIO-eYFP) was added. Roughly a month later, mice were subjected to the LID induction protocol (Fig. 9A). The AAVs were expressed well (Fig. 9B), and the ability of the mAChR agonist oxo-M to enhance somatic excitability of iSPNs in ex vivo brain slices was consistently lost (fig. S3), demonstrating the efficacy of M1R deletion by the CRISPR-saCas9 approach.

**Fig. 10.**
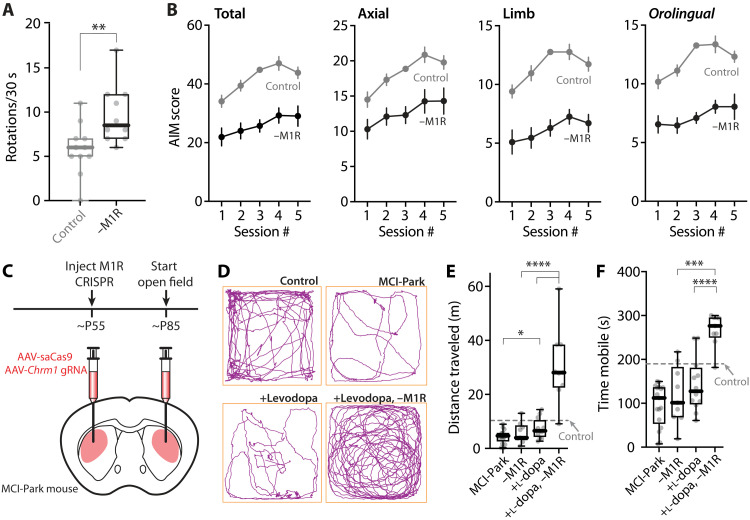
Deletion of M1R from iSPNs attenuated dyskinetic behaviors and enhanced the motoric effect of levodopa. (**A**) Box plot summary of the number of contralateral rotations (in 30 s) recorded 40 min after the fifth levodopa administration (control: *n* = 11 animals; M1R deletion: *n* = 10 animals). (**B**) Plots of total, axial, limb, and orolingual AIM scores as a function of sessions in mice without or with iSPN-specific deletion of M1R. Genetic deletion of M1R in iSPNs produced an overall reduction in AIM scores (control: *n* = 11 animals; M1R CRISPR: *n* = 10 animals; data are mean ± SEM). Total AIM score: time *P* < 0.001, *F*(3.313,62.95) = 22.18; group *P* < 0.001, *F*(1,19) = 29.30. Axial score: time *P* < 0.001, *F*(2.562,48.67) = 21.80; group *P* = 0.0015, *F*(1,19) = 13.80. Limb score: time *P* < 0.001, *F*(3.144,59.74) = 17.01; group *P* < 0.001, *F*(1,19) = 41.43. Orolingual score: time *P* < 0.001, *F*(3.595,68.31) = 12.42; group *P* < 0.001, *F*(1,19) = 44.58. Repeated-measures two-way ANOVA followed by post hoc Bonferroni test. (**C**) Top: The experimental timeline for M1R CRISPR expression and open field experiments with MCI-Park mice. Bottom: Schematic illustrating bilateral injection of M1R CRISPR into the DLS of MCI-Park mice. (**D**) Representative traces of locomotor activity in the open field test. (**E** and **F**) Box plots of distance traveled (E) and time mobile (F) in 5 min (control: *n* = 12 mice; MCI-Park: *n* = 17 mice; MCI-Park with M1R deletion: *n* = 9 mice; MCI-Park with levodopa: *n* = 12 mice; MCI-Park with both M1R deletion and levodopa: *n* = 9 mice). *P < 0.05, ***P < 0.001, and ****P < 0.0001; Mann-Whitney test.

To determine the cellular consequences of M1R deletion after LID induction, we studied visually identified iSPNs in ex vivo brain slices with patch clamp and 2PLSM approaches. As predicted from our findings using systemic administration of the M1R antagonist (Fig. 6, B and C), genetic deletion of M1Rs from iSPNs did not alter the enhancement of somatic excitability observed in the off-state after LID induction (Fig. 9, C and D). Because the emission spectrum of FR and eYFP overlapped with those of the Ca^2+^ dyes, the bAP invasion experiments described above could not be performed. Nevertheless, the dendritic architecture of iSPNs was examined by 2PLSM optical sectioning (Fig. 9, E and F). In agreement with the experiments using systemic M1R antagonists or genetic deletion of CDGI, the apparent increase in iSPN spine density in the off-state following LID induction was blunted by genetic deletion of M1Rs (*P* = 0.0005) (Fig. 9, E and F). Behavioral analysis of mice in which M1Rs had been deleted selectively from iSPNs yielded results that were similar to those obtained from CDGI KO mice. First, the therapeutic benefit of levodopa (as assessed by contralateral rotations) was higher in mice lacking M1Rs in iSPNs (*P* = 0.0056 at the fifth session) (Fig. 10A and movie S2). Second, axial, limb, and orolingual AIMs were markedly attenuated by deletion of M1Rs in iSPNs (Fig. 10B and movie S2). These results, and those described above, argue that dendritic M1R/CDGI signaling in iSPNs contributes to structural and functional adaptations that cause the striatal pathophysiology underlying LID.

Although the number of drug-induced contralateral rotations has been widely used as an assay for determining the potential efficacy of an antiparkinsonian pharmacological treatment (*79*–*82*), there are concerns about its specificity (*83*, *84*). To provide an alternative assay for the therapeutic effect of levodopa, we examined the impact of M1R deletion in a bilateral, genetic model of PD. Specifically, MCI-Park mice [*Dat-cre*^+/−^ × *Ndufs2^fl/fl^* (*85*)] were bilaterally injected with AAVs carrying saCas9 and M1R gRNA expression constructs at ~P55 (Fig. 10C). Four weeks later, mice were examined in an open field. Consistent with previous work (*85*), at this age (~P85), MCI-Park mice were parkinsonian with markedly reduced distance traveled and time spent moving (control versus MCI-Park; *P* < 0.0001) (Fig. 10, D to F). M1R deletion alone did not ameliorate this deficit [MCI-Park versus MCI-Park with M1R deletion (“–M1R”); *P* = 0.6340 and 0.4657 in Fig. 10, E to F, respectively]. A low, nondyskinesiogenic dose of levodopa (1.5 mg/kg) only moderately improved locomotor activity [MCI-Park versus MCI-Park with levodopa (“+l-dopa”); *P* = 0.0339 and 0.1068 in Fig. 10, E to F]. However, with striatal M1R deletion, the same low dose of levodopa profoundly increased locomotion (+l-dopa versus “+l-dopa, −M1R”: *P* < 0.0001 for both distance traveled and time mobile) (Fig. 10, D to F), confirming that suppressing M1R signaling increased the motoric benefit of levodopa.

## DISCUSSION

There are three main conclusions that can be drawn from the data presented. First, in both dSPNs and iSPNs, there was a profound oscillation in both intrinsic excitability and synaptic connectivity between on- and off-states following induction of LID in a mouse model of PD. These cellular and circuitry changes were complementary in nature, reinforcing the idea that an imbalance in the activity of dSPN and iSPN ensembles contributed to LID severity (fig. S4). Second, in parkinsonian and off-state dyskinetic mice where striatal DA levels were low, there was a marked elevation in evoked ACh release, reflecting the loss of inhibitory D2R signaling. Nevertheless, the ability of D2Rs to inhibit ACh release remained intact, arguing that the oscillation in striatal DA concentrations associated with LID on- and off-states was mirrored by a counteroscillation in ACh concentration. Third, cholinergic signaling contributed in a substantial way to the LID-associated adaptations in iSPNs. Blunting M1R or CDGI signaling in iSPNs not only attenuated the oscillations in dendritic excitability and synaptic strength but also had clear behavioral effects—increasing the symptomatic benefit of levodopa and attenuating on-state dyskinesia.

### LID was associated with cell- and state-specific adaptations, particularly in dendrites

Although previous studies have shown that LID induction is accompanied by physiological and anatomical changes in SPNs, most of these studies have focused on alterations that are evident in the off-state, long after the last levodopa dose (*41*–*44*). An implicit assumption of many of these studies is that there are unlikely to be major changes in the relatively short period (hours) after levodopa treatment. Our findings show that this assumption is incorrect.

In dSPNs recorded in ex vivo brain slices taken from mice shortly after their last dose of levodopa (on-state), somatic and dendritic excitability were significantly greater than in dSPNs recorded in slices taken from off-state mice. This observation is not unexpected given the ability of DA acting at D1Rs to increase the excitability of dSPNs at membrane potentials near spike threshold (*48*–*51*). The augmentation in somatic excitability did not readily reverse with antagonism of D1Rs. Although this observation is consistent with the possibility that D1R-mediated modulation of dSPN somatic excitability can persist for an extended period after cessation of receptor stimulation (*54*), further work will be necessary to nail down whether other mechanisms are involved. By contrast, the elevation in dendritic excitability was more time locked to D1R signaling, as it was acutely reversed by an antagonist. Why there is this difference in offset kinetics is unclear at this point but it is likely to involve the regulation of protein phosphatases that reverse the covalent modifications to proteins achieved by D1R activation of PKA (*50*).

Accompanying these changes in excitability were state-dependent alterations in synaptic function. As previously reported, the density of 2PLSM-visible spines on dSPN dendrites fell with LID induction and termination of levodopa (*41*). The mechanisms driving this synaptic change are unclear. What is known is that if levodopa treatment is withheld for several months after 6-OHDA lesioning, then 2PLSM estimates of dSPN spine density eventually fall (*59*). Given that ChIs are disinhibited under this condition, this shift could reflect the sustained engagement of M4R-dependent LTD. The ability of intermittent levodopa treatment to precipitate a similar synaptic attenuation could reflect network activation of disinhibited ChIs and more robust LTD induction (*20*, *86*). Regardless, right after levodopa treatment, the density of detectable spines, particularly those with a mushroom morphology, rose in dSPNs—as might be predicted by engagement of D1Rs and the induction of LTP at axospinous, glutamatergic synapses (*57*, *58*). Consistent with this inference, the amplitude of cortically evoked Sr^2+^-oEPSCs rose during the on-state. Both of these measures of synaptic strength reversed in the off-state, suggesting that the loss of dopaminergic signaling led to depotentiation or LTD (*20*, *44*). The frequency of Sr^2+^-oEPSCs did not change between states, suggesting that there was not a notable change in the number of synapses between on- and off-states. From a network perspective, this drug-induced oscillation in synaptic strength was not driven by the usual factors governing DA release and engagement of D1Rs, such as action initiation or reward. This observation suggests that there is a progressive randomization of corticostriatal synaptic strength with repeated levodopa treatment. If information that enables striatal dSPN ensembles to promote contextually appropriate action is stored in the strength of corticostriatal synapses, then the induction of LID should substantially degrade this information and the ability to move in a purposeful manner (*87*, *88*).

As with dSPNs, the properties of iSPNs in dyskinetic mice shifted between on- and off-states. In the on-state, somatic and dendritic excitability of iSPNs were lower than in the off-state. Unexpectedly, the suppression of iSPN excitability in the on-state did not readily reverse with D2R antagonism. The mechanisms responsible for this persistence, as well as its eventual reversal in the off-state, are unclear. One possibility is that on-state D2R signaling produces long-lasting alterations in ion channels governing excitability [i.e., intrinsic plasticity (*89*, *90*)]. In iSPNs, D2Rs could bring about this modulation through activation of phospholipase C and the protein phosphatase calcineurin, which regulates phosphorylation of a variety of ion channels, including Ca_v_1 Ca^2+^ channels (*91*). By contrast, the reasons for the elevation in off-state excitability of iSPNs are easier to surmise. Loss of ambient D2R signaling and disinhibition of ChIs undoubtedly contributed to this shift (*22*–*24*, *92*). Notably, pharmacological antagonism of M1Rs or genetic deletion of M1Rs/CDGI blunted the off-state shift in dendritic excitability.

Another state-dependent change in iSPNs was in the strength of corticostriatal glutamatergic synapses. The amplitude of optogenetically evoked, asynchronous Sr^2+^-oEPSC in iSPNs fell during the on-state and rose during the off-state, consistent with the well-described role of D2Rs in the modulation of long-term synaptic plasticity in these cells (*57*, *64*, *93*, *94*). The frequency of asynchronous Sr^2+^-oEPSCs did not change between these states, arguing that the total number of synapses was unchanged. Although the augmentation of synaptic strength in the off-state was consistent with previous studies (*42*), the apparent stability in number of synapses was not. Previously, it was reported that iSPN spine density—and by inference synaptic density—increased in the off-state after the induction of dyskinesia (*41*, *43*, *67*). Although our estimates of spine density using 2PLSM rose in the off-state, reaching values close to those seen before 6-OHDA lesioning, higher-resolution confocal imaging of iSPN dendrites uncovered only a modest change in spine density, well below values seen in controls. Together, our findings suggest that there is an alternative interpretation of the data. With the best optical approaches, the density of SPN spines in proximal dendrites is roughly 2.6 spines/μm, with many spines being less than a half micrometer in diameter (*68*, *69*). Using confocal microscopy with a high-NA lens (1.49), proximal spine density was estimated to be about 2 spines/μm, suggesting that about a quarter of the spines were being missed. Using 2PLSM with a lower NA lens (0.9), proximal spine density was estimated to be about 1.3 spines/μm, suggesting that roughly half of the spines were not being detected. The most parsimonious interpretation of the imaging and physiology is that what is changing between on- and off-states is not the number of spines but rather their diameter and resolvability with optical methods. When the confocal analysis was limited to large spines (>0.4-μm diameter) (fig. S5), there was good alignment with the 2PLSM data. This is an important point because it suggests that iSPNs in an adult brain are not breaking and forming new synapses but rather are modulating their strength (which results in a size change). It is still the case that this modulation in synaptic strength is uncoupled from actions and their outcomes, unlike the situation in the normal striatum. As noted above, this should result in a degradation in synaptic information and an impaired ability to properly coordinated movement.

Despite its limitations, 2PLSM remains an important tool for detecting changes in structural connectivity. The size and morphology of dendritic spines are strongly correlated with their synaptic function (*65*, *95*, *96*). In particular, larger spines are associated with larger synaptic currents, whereas smaller spines (and filopodia; diameter < 0.3 um) are associated with smaller or undetectable currents (“silent synapse”). Therefore, 2PLSM provides an important window into synaptic function.

Repeated administration of DA-mimetics, which, such as levodopa, can markedly elevate extracellular DA, also leads to alterations in SPN spine density and neuronal excitability (*97*, *98*). For instance, repeated cocaine exposure increases SPN spine density and decreased their intrinsic excitability (*99*, *100*). However, there are several differences with the LID condition. First, cocaine-induced alterations in dSPN spines and miniature excitatory postsynaptic current frequency (but not amplitude) (*101*) were suggestive of an increase in synapse number rather than strengthening of existing synapses. Second, the increase in spine density induced by repeated cocaine administration was stable for days after termination of drug treatment (*99*, *101*). Last, dSPNs were more sensitive to cocaine administration than iSPNs (*101*, *102*), in contrast to the situation described here following LID induction.

### Dysregulation of ChIs contributed to LID severity

In the healthy striatum, the interaction between DA and ACh release by ChIs is important in modulating the activity of SPNs and motor control (*15*, *103*). In both dSPNs and iSPNs, this interaction is largely antagonistic, which creates a means of dynamically regulating basal ganglia output. In iSPNs, DA activation of D2Rs diminishes somatodendritic excitability and promotes LTD at glutamatergic synapses, whereas ACh activation of M1Rs does the opposite; in dSPNs, D1Rs promote somatodendritic excitability and LTP at glutamatergic synapses, whereas M4Rs do the opposite (*20*, *23*, *24*, *49*, *57*, *91*, *92*). Complementing this interaction at the level of SPNs, ACh triggers DA release by activating nicotinic receptors on dopaminergic terminals (*104*–*106*), whereas dopaminergic activation of D2Rs expressed by ChIs inhibits the autonomous spiking of ChIs and ACh release from axon terminals (*15*–*18*, *28*).

In PD, this dynamic interaction is disrupted. The loss of the striatal dopaminergic innervation in patients with PD has long been thought to disinhibit ChIs, leading to a hypercholinergic state, a persistent imbalance in the excitability of iSPNs and dSPNs and ultimately bradykinesia (*1*, *21*, *28*–*34*). Our experiments using the genetically encoded optical sensor of ACh (GRAB_ACh3.0_) are consistent with this hypothesis, showing that in the 6-OHDA–lesioned striatum, electrically evoked ACh release is significantly greater than in control striata. This up-regulation was primarily attributable to the loss of presynaptic D2R signaling, as the D2R agonist quinpirole was able to almost eliminate evoked ACh release. That said, the fact that peak ACh release was significantly greater in the lesioned striatum than in the control striatum in the presence of the D2R antagonist sulpiride is consistent with previously reported impairment in mAChR autoreceptor function in DA-depleted striatum (*28*).

The role of elevated ACh release by ChIs in parkinsonism is widely accepted and consistent with the clinical utility of mAChR antagonists, but the involvement of ChIs in LID has been controversial (*35*–*37*). The issue in the field is not whether they influence LID severity but rather how. Several lines of study suggest that hyperactivity of ChIs exacerbates LID severity. For example, ablation or chemogenetic inhibition of ChIs lessens the severity of LID in rodent models (*39*, *40*), as does genetic perturbation of ChIs (*107*). On the other hand, enhancing M4R signaling in dSPNs attenuates LID induction (*20*, *38*), and genetic deletion of D5 DA receptors from ChIs, which appears to lower their excitability, worsens LID (*108*). Similarly, as shown here, deleting M1Rs in iSPNs attenuated LID induction, as did deletion of its downstream signaling partner, CDGI.

Much of this controversy may stem from a failure to distinguish between what is happening in on- and off-states. Our GRAB_ACh3.0_ experiments show that DA continues to robustly inhibit ACh release from ChIs in the striatum of dyskinetic mice. As a consequence, it seems very unlikely that when striatal DA levels rise during the on-state that ChI ACh release rises in parallel, regardless of whether there are modest increments in ChI intrinsic excitability induced by repeated levodopa treatment (*86*, *109*). By contrast, during the off-state, when striatal DA levels plummet, ChIs are undoubtedly disinhibited. Off-state ACh release by ChIs should also be augmented by an elevation in intrinsic excitability induced by repeated levodopa treatment (*86*, *109*). The elevation in the dendritic excitability of iSPNs in the off-state is precisely what would be predicted by an up-regulation in ACh release and activation of M1Rs (*22*–*24*, *92*). The analysis of SPN synaptic plasticity also aligned with this hypothesis; that is, during the off-state, the strength of iSPN glutamatergic synapses rose, whereas synaptic strength in dSPNs fell. These synaptic shifts are consistent with the well-described effects of ACh (and DA) on synaptic plasticity in these two cell types (*9*, *10*, *35*, *71*, *72*). This functional plasticity was mirrored structurally, as the number of spines discernible with 2PLSM rose in iSPNs and fell in dSPNs during the off-state.

More direct evidence for the conclusion that ChIs play an important role in the pathophysiology underlying LID came from the demonstration that disrupting M1R signaling in iSPNs, either by deleting CDGI globally or by CRISPR-saCas9–mediated deletion of *Chrm1* specifically from them, blunted the induction of LID. Our results bring specificity to these effects by demonstrating that the engagement of ChIs and their remodeling of striatal circuitry are occurring primarily during the off-state. The importance of off-state striatal adaptations is underscored by recent work showing that during this period, iSPNs up-regulate the expression of GluN2B-containing *N*-methyl-d-aspartate (NMDA) receptors, which enable the induction of LTP (*63*). This observation is consistent with the off-state strengthening of corticostriatal synapses and spine enlargement in iSPNs reported here. Furthermore, the GluN2B up-regulation is necessary for not just the induction of LID but its expression after repeated levodopa treatment (*63*).

What is less clear is how the off-state adaptations in iSPNs promote dyskinesia with the next dose of levodopa. Previous work has shown that enhancing the intrinsic excitability of iSPNs in the on-state attenuates LID (*62*). Hence, had the elevation in off-state iSPN excitability persisted into the on-state, it would have been antidyskinetic. However, this alteration was not persistent and reversed after levodopa treatment. As discussed above, the mechanisms responsible for the reversal remain to be determined. Nevertheless, there are at least two potential “carry-over” mechanisms.

The first possibility is that aberrant off-state learning disrupts iSPN ensemble activity in the on-state. Increased excitability in the off-state, particularly in dendrites, will enhance the induction of postsynaptic LTP in iSPNs, which depends upon depolarization and NMDA receptor opening. Deletion of LTP-promoting M1Rs or their dendritic signaling partner CDGI (*22*) blunted the off-state strengthening of corticostriatal synapses. How might the induction of synaptic potentiation in iSPNs during the off-state promote LID? Appropriately timed and coordinated movement requires coactivation of iSPN and dSPN ensembles. The architecture of SPN ensembles is largely dependent upon how the activity in cortical ensembles is translated by striatal circuits. There are a variety of factors that contribute to this translation, but the weighting of synaptic inputs by SPNs is certainly an important one. This synaptic weight or strength is normally shaped by experience and repetition (*110*–*112*). However, in the parkinsonian striatum, this linkage is lost. Recent work has underscored the importance of aberrant learning in PD, suggesting that engagement of cortical circuitry associated with a particular action in the absence of striatal DA (and elevation in ACh) contributes to the inability to perform that action in the future (*113*). It is easy to imagine that in this situation, the striatal circuitry “interprets” the neuromodulator imbalance as a negative outcome, leading to strengthening of connectivity between action-associated cortical circuits and iSPN ensembles while weakening those with dSPN ensembles. This kind of aberrant, outcome-independent striatal learning could also be happening in the off-state. If this were the case, then the identity of the cortical synapses being strengthened would depend upon which cortical ensembles were active in the off-state, as coincident pre- and postsynaptic activity is necessary for LTP induction (*57*). The identity of these ensembles is uncertain, especially given the profoundly hypokinetic nature of the off-state. Conversely, the sustained elevation in striatal DA and drop in ACh during the on-state also create an aberrant learning signal in the striatum, akin to a positive outcome for any and all actions, promoting corticostriatal LTD in iSPNs (*57*, *64*, *93*, *94*). Repeated cycles of aberrant learning could scramble the corticostriatal synaptic architecture and ensemble activity, leading to progressively abnormal, meaningless movement.

The second possibility is that aberrant off-state activity changes in the induction threshold for synaptic plasticity in the on-state (*114*–*116*). The Bienenstock, Cooper, and Munro (BCM) model posits that the induction threshold for LTP and LTD is determined by past neural activity. In the case of LID, a period of high iSPN activity in the off-state might increase the threshold for LTP induction and promote pathophysiological LTD induction in the on-state, whereas a period of low dSPN activity in the off-state might promote LTP and blunt LTD induction in the on-state. This combination could drive the imbalance in iSPN and dSPN ensemble structure thought to play an integral role in LID (*62*, *117*).

### Translational implications

At present, the strategies for alleviating LID in patients with PD are very limited. Our studies demonstrate that suppressing M1R/CDGI signaling in iSPNs alleviates LID while enhancing the symptomatic benefits of levodopa in rodent models of PD. Global antagonism of M1Rs would come with unacceptable side effects. The best translational path forward for this observation would involve a regionally targeted gene therapy. The recent development of enhancer sequences that can be used to limit gene expression specifically to iSPNs in the dorsal striatum (*118*) creates a potential path forward for a novel gene therapy targeting M1Rs or CDGI that does not rely upon neurosurgical approaches.

## MATERIALS AND METHODS

### Animals

Animal use procedures were approved by the Northwestern Institutional Animal Care and Use committee (IS00032011, IS00017034, and IS00015064). Male C57Bl/6 hemizygous mice (7 to 12 weeks of age) expressing tdTomato or eGFP under control of *Drd1a* or *Drd2* regulator elements (RRID: MMRRC_030512-UNC and MMRRC_000230-UNC; backcrossed to C57BL/6 background) were used. In some experiments, these mice were crossed with CDGI KO mice in a C57BL/6J background originally generated at Massachusetts Institute of Technology (MIT) (*22*, *25*). BAC transgenic mice expressing Cre recombinase under control of the *Adora2a* regulatory elements (RRID: MMRRC 031168-UCD) were used for high-resolution confocal microscopy and M1R CRISPR experiments. MCI-Park mice [*Dat-cre*^+/−^ × *Ndufs2^fl/fl^*, RRID: IMSR_JAX:036313; (*85*)] of both sexes and their littermate controls (*Dat-cre*^−/−^ × *Ndufs2^fl/fl^*) were used for the open field test.

### Unilateral 6-OHDA model of PD and LID

Mice were anesthetized using an isoflurane precision vaporizer (at 5% isofluorane during induction and 2% isofluorane during maintenance phase) and positioned in a stereotaxic frame (David Kopf Instruments, model 940). Mice were administered analgesics meloxicam [0.1 mg/kg, subcutaneously (sc); METACAM, Covetrus] before surgery. After the skin and fascia were retracted to expose the skull, a small hole was drilled over the MFB. Free base 6-OHDA hydrochloride (3.5 mg ml^−1^; Sigma-Aldrich, catalog no. H4381) freshly dissolved in saline with 0.02% l-ascorbic acid (Sigma-Aldrich, catalog no. A92902) was injected using a glass calibrated micropipette (Drummond Scientific Company, Broomall, PA, 200005) at the following coordinates: anterior-posterior (AP) (relative to Bregma): –0.7 mm; medial-lateral (ML): –1.2 mm; dorsal-ventral (DV): −4.75 mm (from dura). Mice were monitored daily post-op and supplemented with saline injections and high fat/high sucrose food as needed (*63*). Three to 4 weeks after surgery, the degree of lesioning of nigrostriatal DA neurons was assessed with a drug-free cylinder test (*41*, *63*). Within 1 to 7 days of the cylinder test, mice underwent behavioral testing for AIMs as previously described (*4*, *41*, *63*). Briefly, mice were transferred to a behavioral testing room, placed in clean cage bottoms without bedding, and administered intraperitoneal injections of l-dopa at 6 mg/kg for the first two sessions and 12 mg/kg for the later sessions. Benserazide was coadministered at 12 mg/kg to inhibit peripheral conversion of levodopa to DA. Behavioral testing was performed every other day for a total of five test sessions. AIMs (axial, limb, and orolingual movements) were scored by an experimenter blinded to the genotype/treatment groups using an established protocol (*4*, *41*, *63*): Abnormal axial, limb, and orolingual behaviors were observed for 1 min every 20 min and rated on a scale from 0 to 4 for each parameter on the basis of duration and continuity. Mice were euthanized for ex vivo experiments 24 to 48 hours (off-state) or 0.5 hour (on-state) after the last levodopa administration. For ex vivo experiments of on-state mice, the slices were assessed by physiology or 2PLSM for no more than 4 hours after l-dopa administration. Striatal sections from a subset of mice were immunostained for tyrosine hydroxylase to verify successful lesion.

### Immunohistochemistry and confocal microscopy

Anesthetized mice were perfused transcardially with saline briefly (~1 min) and then with ice-cold 4% (wt/vol) paraformaldehyde (PFA) (Electron Microscopy Sciences, catalog no. 15700) in 1× phosphate-buffered saline (PBS) (Sigma-Aldrich, catalog no. 79383). Brains were dissected out and postfixed in 4% PFA-PBS overnight at 4°C. Coronal slices (100-μm-thick) were cut using a vibratome (Leica VT1200, Leica Biosystems, Germany), permeabilized, and blocked at 4°C in PBS including 5% normal goat serum (Gibco, catalog no. 16-210-072) and 0.2% Triton-X 100 (Sigma-Aldrich, catalog no. X100) (NGS/PBST), incubated with rabbit pERK antibody (Cell Signaling Technology, #9101; 1:100 dilution) and mouse tyrosine hydroxylase antibody (Immunostar, #22941; 1:200 dilution) diluted in NGS-PBST overnight at 4°C. After four washes in NGS/PBST, the slices were incubated with 1:1000 goat anti-rabbit Alexa Fluor 488 or goat anti-mouse Alexa Fluor 555 (Invitrogen, A-11001 and A-21428) for 2 hours at room temperature. Slices were washed four times with NGS-PBST and once with PBS, mounted with VECTASHIELD Antifade Mounting Medium (Vector Laboratories, H-1000-10), and viewed under an automated laser scanning confocal microscope (FV10i-DUC, Olympus). Images were adjusted for brightness, contrast, and pseudocoloring in Fiji (ImageJ, US National Institutes of Health).

### Viral injections

To study synaptic responses at corticostriatal synapses, 0.15 μl of AAV5-hSyn-hChR2(H134R)-eYFP (Addgene, #26973) was injected into the M1 motor cortex ipsilateral to the lesion site at the following coordinate (relative to Bregma): ML: -1.60 mm, AP: 1.15 mm, and DV: 1.55 mm. To sparsely label iSPNs for high-resolution confocal imaging of dendritic spines, *Adora2*-Cre mice (~2 months old) were injected with AAV9-pCAG-flex-eGFP-WPRE (Addgene, #51502) (0.450 μl; titer of 5 × 10^10^ viral genome/ml) into the left DLS at the following coordinate (relative to Bregma): AP: 0.7 mm, ML: –2.35 mm, and DV: ~3.35 mm. To express the ACh sensor GRAB_ACh3.0_ in the striatum, 0.6 μl of AAV5-hSyn-ACh3.0(ACh4.3) (WZ Biosciences, YL001003-AV5) was injected into the DLS ipsilateral to the 6-OHDA lesion at the following coordinate (relative to Bregma): ML: –2.2 mm, AP: 0.8 mm, and DV: 3.3 mm. For M1R gene deletion in 6-OHDA–lesioned mice using CRISPR-saCas9, mice with successful 6-OHDA lesions were randomly assigned to receive viral injection of either a mixture of two viruses (at 1:1 ratio): AAV9-hSyn-DIO-saCAS-minWPRE and AAV9-U6-gRNA(M1R)-U6-gRNA2-hSyn-FR (M1R CRISPR; custom made by Virovek) or a mixture of saline and the gRNA virus (control). The viral injection (0.5 μl) was in the DLS (ipsilateral to the 6-OHDA lesion) at a total of six sites at the following coordinates (relative to Bregma): AP: 0.9 mm, ML: –2.3 mm, DV: −3.4 mm, and −2.8 mm; AP: 0.6 mm, ML: –1.5 mm, DV: −3.4 mm, and −2.8 mm; AP: 0.24 mm, ML: –1.9 mm, and DV: −3.3 and −2.7 mm. For ex vivo experiments that required identification of CRISPR-expressing iSPNs, a third virus: AAV9-EF1a-DIO-eYFP (Addgene, #27056) was mixed with M1R CRSIPR or control (at 1:1:1 ratio) and injected. For M1R gene deletion in MCI-Park mice, ~P55 mice received bilateral injections (0.5 μl per site) of a mixture of AAV9-hSyn-saCAS-minWPRE and AAV9-U6-gRNA(M1R)-U6-gRNA2-hSyn-FR (at 1:1 ratio; both custom made by Virovek) at the following coordinates (relative to Bregma): AP: 0.9 mm, ML: ±2.3 mm, DV: −3.4 and −2.8 mm; AP: 0.6 mm, ML: ±1.5 mm, DV: −3.4 and −2.8 mm; AP: 0.24 mm, ML: ±1.9 mm, DV: −3.3 and −2.7 mm*.* All experiments were performed about 3 to 4 weeks (for Cre-independent expression) or 4 to 5 weeks (for Cre-dependent expression) after viral injections.

### Slice electrophysiology

Mice were deeply anesthetized with a mixture of ketamine (100 mg/kg) and xylazine (7 mg/kg) and perfused transcardially with ice-cold sucrose-based cutting solution containing 181 mM sucrose, 25 mM NaHCO_3_, 1.25 mM NaH_2_PO_4_, 2.5 mM KCl, 0.5 mM CaCl_2_, 7 mM MgCl_2_, 11.6 mM sodium ascorbate, 3.1 mM sodium pyruvate, and 5 mM glucose (305 mosmol/liter). Sagittal slices (280-μm-thick) were sectioned using a vibratome (Leica VT1200). After cutting, slices were incubated at 34°C for 30 min in artificial cerebrospinal fluid (ACSF) containing 124 mM NaCl, 3 mM KCl, 1 mM NaH_2_PO_4_, 2.0 mM CaCl_2_, 1.0 mM MgCl_2_, 26 mM NaHCO_3_, and 13.89 mM glucose, after which they were stored at room temperature until recording. External solutions were oxygenated with carbogen (95% CO_2_/5% O_2_) at all time.

Individual slices were transferred to a recording chamber and continuously superfused with ACSF (2 to 3 ml/min at room temperature). D1-Tdtomato– or D2-eGFP–expressing SPNs in the striatum were first identified with an Olympus BX-51–based two-photon laser scanning microscope (Ultima, Bruker, Billerica, MA). Whole-cell patch clamp was then performed in identified SPNs, aided by visualization with a 60×/0.9 NA water-dipping objective lens and a ^1^/_2_-inch (12.7-mm) charge-coupled device video camera (Hitachi) imaged through a Dodt contrast tube and a 2× magnification changer (Bruker). For somatic, dendritic, and morphological experiments, patch pipettes (3- to 4-megohm resistance) were loaded with internal solution containing 115 mM k-gluconate, 20 mM KCl, 1.5 mM MgCl_2_, 5 Hepes, 2 mM Mg–adenosine 5′-triphosphate (ATP), 0.5 mM Na–guanosine 5′-triphosphate (GTP), and 10 mM Na-phosphocreatine (pH 7.25 and osmolarity of 280 to 290 mosmol/liter), supplemented with 100 μM Fluo-4 (Thermo Fisher Scientific, F14200) and 50 μM Alexa Fluor 568 hydrazide (Thermo Fisher Scientific, A10437). Cells were recorded in the current-clamp configuration. For Sr^2+^-oEPSC experiments, patch pipettes (3- to 4-megohm resistance) were loaded with 120 mM CsMeSO_3_, 5 mM NaCl, 0.25 mM EGTA, 10 mM Hepes, 4 mM Mg-ATP, 0.3 mM Na-GTP, 10 mM tetraethylammonium, and 5 mM QX-314 (pH 7.25 and osmolarity of 280 to 290 mosmol/liter). SPNs were held at −70 mV in the voltage-clamp configuration. After patching, recording solution was changed to Ca^2+^-free ACSF containing 3 mM SrCl_2_ and 10 μM gabazine (10 μM; to suppress γ-aminobutyric acid type A–mediated currents). Slices were superfused with this Ca^2+^-free solution for 25 min before recording. EPSCs were evoked every 30 s by whole-field LED illumination (single 0.3-ms pulses) using CoolLED p100-470 (CoolLED, Andover, UK). All the electrophysiological recordings were made using a MultiClamp 700B amplifier (Axon Instrument, USA), and signals were filtered at 2 kHz and digitized at 10 kHz. Voltage protocols and data acquisition were performed by Prairie View 5.3 (Bruker). The amplifier command voltage and all light source shutter and modulator signals were sent via the PCI-NI6713 analog-to-digital converter card (National Instruments, Austin, TX).

### Imaging of spines and dendritic Ca^2+^ transients with 2PLSM

After whole-cell recording configuration was established, cells were allowed to equilibrate with dyes for at least 15 min before imaging. The recorded SPN was detected using 810-nm excitation laser (Chameleon Ultra II, Coherent, Santa Clara, USA). Dendritic structure was studied by imaging the red signal of Alexa Fluor 568 detected by a Hamamatsu R3982 side-on photomultiplier tube (PMT) (580 to 620 nm; Hamamatsu Photonics, Japan). Calcium transients, as signals in the green channel, were detected by a Hamamatsu H7422P-40 GaAsP PMT (490 to 560 nm). Signals from both channels were background subtracted before analysis. Line scan signals were acquired at 128 pixels per line resolution and 10 μs per pixel dwell time along a dendritic segment. Ca^2+^ signals were quantified as the area of increase in green fluorescence from baseline normalized by the average red fluorescence (ΔG/R). The dendritic excitability index was calculated as the ratio of the Ca^2+^ signal from a distal location to the Ca^2+^ signal from a proximal location on the same dendrite. Only data with similar baseline levels (G_0_/R_0_) for proximal and distal locations were included.

For assessment of dendritic spine density, images of dendritic segments (proximal: 40 to 60 μm from soma; distal: >80 μm from soma) were acquired with 0.15-μm pixels with 0.3-μm *z*-steps. Images were deconvolved in AutoQuant X3.0.4 (Media Cybernetics, Rockville, MD), and semiautomated spine counting was performed using 3D reconstructions in NeuronStudio (CNIC, Mount Sinai School of Medicine, New York, NY). On average, two proximal and two distal dendrites were imaged and analyzed per neuron.

### Two-photon imaging of ACh sensor

ACh release was assessed by imaging GRAB_ACh3.0_, a genetically encoded fluorescent sensor of ACh (*73*), using 2PLSM. Acute slices with striatal expression of GRAB_ACh3.0_ were prepared as described above, transferred to a recording chamber, and continuously perfused with normal ACSF at 32° to 34°C. A two-photon laser (Chameleon Ultra II, Coherent, Santa Clara, CA) tuned to 920 nm was used to excite GRAB_ACh3.0_. Fluorescence was imaged using an Ultima In Vitro Multiphoton Microscope system (Bruker) with an Olympus 60×/0.9 NA water-immersion objective lens and a Hamamatsu H7422P-40 GaAsP PMT (490 to 560 nm). Time-series images of the GRAB_ACh3.0_ were acquired with 0.388 μm by 0.388 μm pixels, 10-μs dwell time, and a frame rate of 5.01 frames per second (fps) (21.26 fps for sample traces). After a 10-s baseline acquisition, synchronous ACh release was evoked by delivering a single (1 ms × 0.3 mA) or a train of 20 electrical stimuli (1 ms × 0.3 mA at 20 Hz) by a concentric bipolar electrode (CBAPD75, FHC) placed at 200 μm ventral to the region of interest (ROI). Imaging was continued for at least another 5 s. Two trials were performed for each stimulation protocol, and data were averaged. The slices were imaged for (in this sequence) control, 50 nM DA (only for mice with 6-OHDA lesions), 10 μM quinpirole, and 10 μM sulpiride treatment conditions, with at least 5 min of perfusion for each treatment. Sodium metabisulfite (50 μM) was included in the bath solution to slow down DA oxidation. The maximal fluorescence intensities (*F*_max_) were determined by applying 100 μM acetylcholine chloride (to saturate GRAB_ACh3.0_ signal). The minimal fluorescence intensities (*F*_min_) were determined by applying 10 μM tetrodotoxin (to block any basal transmission) or from quinpirole-treated baselines, whichever was smaller. A circle with a diameter of 50 μm in the center of the imaging field was the ROI used for analysis. Fluorescent intensity data were analyzed by custom Python code (www.doi.org/10.5281/zenodo.14163731). Briefly, the fluorescence intensity values were first background subtracted (the background resulted from PMT was measured by imaging with same PMT voltage but zero laser power). Baseline fluorescence *F*_0_ was the average fluorescence over the 1-s period right before stimulation. Δ*F* = *F* – *F*_0_ was normalized by (*F*_max_ − *F*_min_) and then analyzed.

### High-resolution confocal microscopy of sparsely labeled neurons

*Adora2*-Cre mice injected with AAV9-pCAG-flex-eGFP-WPRE and treated with four different conditions (control, 6-OHDA, LID off-state, and LID on-state) were anesthetized with isoflurane, followed by ketamine/xylazine mixture. LID on-state mice received a sixth dose of levodopa [12 mg/kg, supplemented with benserazide (12 mg/kg)] 1 hour before anesthesia. The mice were transcardially perfused with 1× PBS (~20 ml), followed by 4% PFA-PBS (~30 ml). Brains were then dissected out, postfixed in 4% PFA-PBS for 1.5 to 2 hours, transferred to 1× PBS with 0.1% sodium azide, and stored at 4°C until sectioning. Sixty-micrometer sagittal sections containing the DLS were cut using a Leica VT1200S vibratome. Sections were mounted onto glass slides with no. 1.5 cover glasses using ProLong Diamond Antifade Mountant (Thermo Fisher Scientific, catalog no. P36961).

A Nikon AX R confocal laser microscope (Nikon Instruments Inc., Melville, NY) from the Center for Advanced Microscopy & Nikon Imaging Center at Northwestern University was used to image dendritic spines from sparsely labeled iSPNs in fixed brain sections. The z-stack images were acquired with a 60× oil immersion objective (NA = 1.49) at 0.125-μm intervals with 0.09-μm pixel size. The images were then denoised and deconvolved with Nikon NIS-Elements AR 5.41.02 software. About 30-μm dendritic segments measuring at ~30 μm from the soma were analyzed. Spine density analysis was performed with Imaris 10.0.0 software (Oxford Instruments). Briefly, the dendrite of interest was first isolated by adding a new Surface. Surrounding dendrites and axons were masked by the Surface function. “Labkit for pixel classification” was adopted to train the detection of signals over noises to create a tight surface. The surface was further cut to include only the dendritic segment of interest. Filament for the dendritic segment was then created with the aid of an embedded supervised learning function. Spine detection was subsequently performed with the thinnest spine head set at 0.188 μm and maximum spine length set at 5 μm, and no branched spines were allowed. Spines were further determined with an embedded supervised learning function and manually corrected if necessary.

### Open-field test

MCI-Park mice and their littermate control (~P85) were brought to the red light–lit testing room for 10 to 60 min before the start of each test. Mice were habituated to the open-field box (40 cm by 40 cm by 34.6 cm) for four consecutive days with one trial test per day. Baseline activities of control, MCI-Park, and MCI-Park mice with M1R deletion were quantified from the fourth day. On the fifth day, mice were injected with levodopa (1.5 mg kg^−1^) and benserazide (12 mg kg^−1^) 30 min before testing. Locomotor activity in the open field was monitored during a 5-min test session using ANY-maze (ver. 7.15, Stoelting Co., Wood Dale, IL), and the total distance traveled and time mobile were analyzed.

### Data acquisition and statistical analysis

Electrophysiology and imaging data were acquired using PCI-NI6052E analog-to-digital converter card (National Instruments) and Prairie View 5.3 (Bruker). Off-line analyses of somatic excitability data, dendritic excitability data, and ACh sensor imaging data were performed using custom-written Python scripts (www.doi.org/10.5281/zenodo.14145776; www.doi.org/10.5281/zenodo.14163502; www.doi.org/10.5281/zenodo.14163731). Amplitudes of Sr^2^-oEPSCs were analyzed automatically using TaroTools’ Event Detection in Igor Pro 8 (WaveMetrics, Portland, OR), followed by visual verification. Events were measured between 40 and 400 ms after photostimulation. The threshold for detection of an event was that it be greater than five SD above the noise. All summary data were presented as nonparametric box-whisker plots and statistical analyses performed by Prism 6 or 10 (GraphPad Software Inc., La Jolla, CA). The stated *n* indicates the number of cells (in electrophysiology experiments), the number of ROIs (in ACh sensor imaging), or the number of mice (in behavior tests). Unless otherwise stated, comparisons were made using nonparametric tests (Mann-Whitney test for unpaired and Wilcoxon test for paired data) because the normality of data could not be assumed. Differences with *P* < 0.05 are considered statistically significant. Statistical analysis for AIMs data was performed using parametric repeated-measures two-way analysis of variance (ANOVA), followed by post hoc Bonferroni test.
