# Supplementary material for: State-dependent modulation of spiny projection neurons controls levodopa-induced dyskinesia in a mouse model of Parkinson’s disease
Source: Sci Adv. 2025 Dec 3;11(49):eadv8224. doi: 10.1126/sciadv.adv8224 (PMC12674127; doi:10.1126/sciadv.adv8224)
Supplement: Supplementary file 1 — Figs. S1 to S5 Legends for movies S1 and S2 [file sciadv.adv8224_sm.pdf]

Science Advances
AAAS

Supplementary Materials for

State-dependent modulation of spiny projection neurons controls levodopa-induced dyskinesia in a mouse model of Parkinson's disease

Shenyu Zhai *et al.*

Corresponding author: D. James Surmeier, j-surmeier@northwestern.edu

Sci. Adv. **11**, eadv8224 (2025)
DOI: 10.1126/sciadv.adv8224

The PDF file includes:

Figs. S1 to S5
Legends for movies S1 and S2

Other Supplementary Material for this manuscript includes the following:

Movies S1 and S2

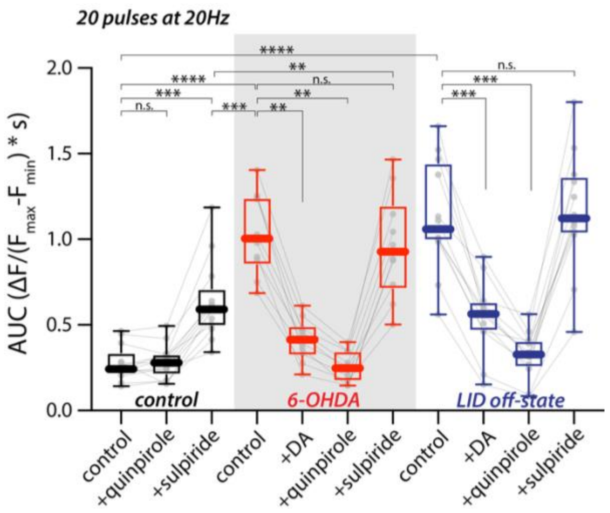

Fig. S1. ACh release evoked by burst stimulation was elevated in 6-OHDA lesioned and LID off-state mice and suppressed by D2R agonists.

Box plot summary of $GRAB_{ACh3.0}$ signals evoked by burst stimulation in unlesioned, 6-OHDA lesioned and LID off-state mice. Same as with single stimulation, ACh release evoked by burst stimulation (20 pulses at 20 Hz) was significantly elevated in 6-OHDA lesioned and LID off-state mice. Bath application of DA (50 nM) or quinpirole (10 μM) strongly suppressed ACh release (unlesioned control, n = 13 ROIs from 3 mice; 6-OHDA, n = 10 ROIs from 3 mice; off-state, n = 11 ROIs from 5 mice). **** $p < 0.0001$, *** $p < 0.001$, ** $p < 0.01$, n.s., not statistically significant, Mann-Whitney test for unpaired data and Wilcoxon for paired data.

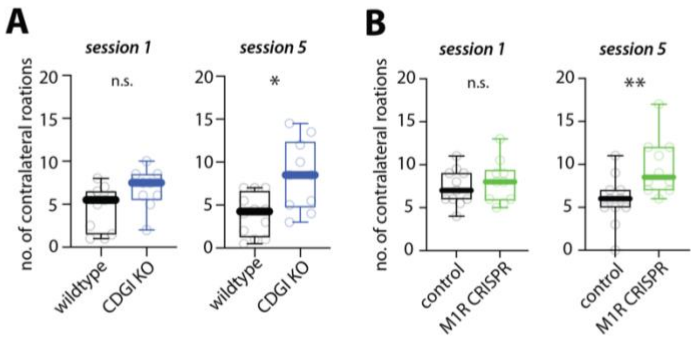

Fig. S2. Genetic perturbation of M1R-CDGI signaling in iSPNs increases the therapeutic effect of levodopa in parkinsonian mice

(**A**) Box plot summary of the number of contralateral rotations (in 30 s) recorded after the first and fifth levodopa administration in wildtype or CDGI KO mice (wildtype n = 11-12 animals; CDGI KO, n = 9-10 animals). The session 5 data were the same as in Fig. 8A. * $p < 0.05$, n.s. not statistically significant, Mann-Whitney test.

(**B**) Box plot summary of the number of contralateral rotations (in 30 s) recorded after the first or fifth levodopa administration in control or M1R CRISPR mice (control n = 11 animals; M1R CRISPR, n = 10 animals). The session 5 data were the same as in Fig. 10A. ** $p < 0.01$, n.s. not statistically significant, Mann-Whitney test.

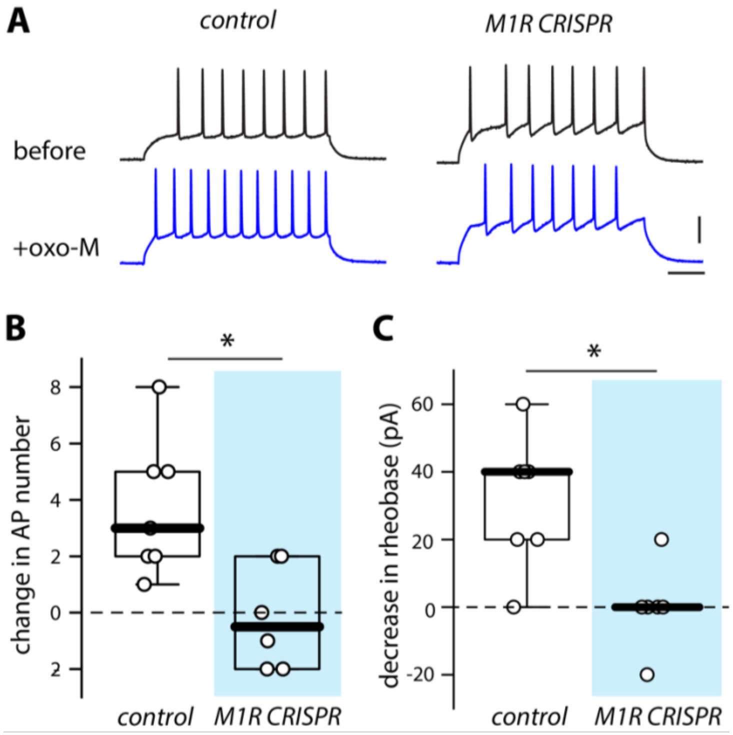

Fig. S3. Functional validation of M1R CRISPR.
(**A**) Example traces of somatic voltage recordings in response to a 180-pA current injection from iSPNs expressing M1R CRISPR or gRNA alone (control) before and after application of oxo-M. Scale bars are 40 mV and 100 ms.
(**B**) Box plot summary of the effect of oxo-M on the number of action potentials (APs) evoked (control, $n = 7$ cells from 3 mice; M1R CRISPR, $n = 6$ cells from 3 mice). The increase in somatic excitability by oxo-M was prevented by M1R CRISPR. * $p < 0.05$, Mann-Whitney test.
(**C**) Box plot summary of the effect of oxo-M on rheobase. * $p < 0.05$, Mann-Whitney test.

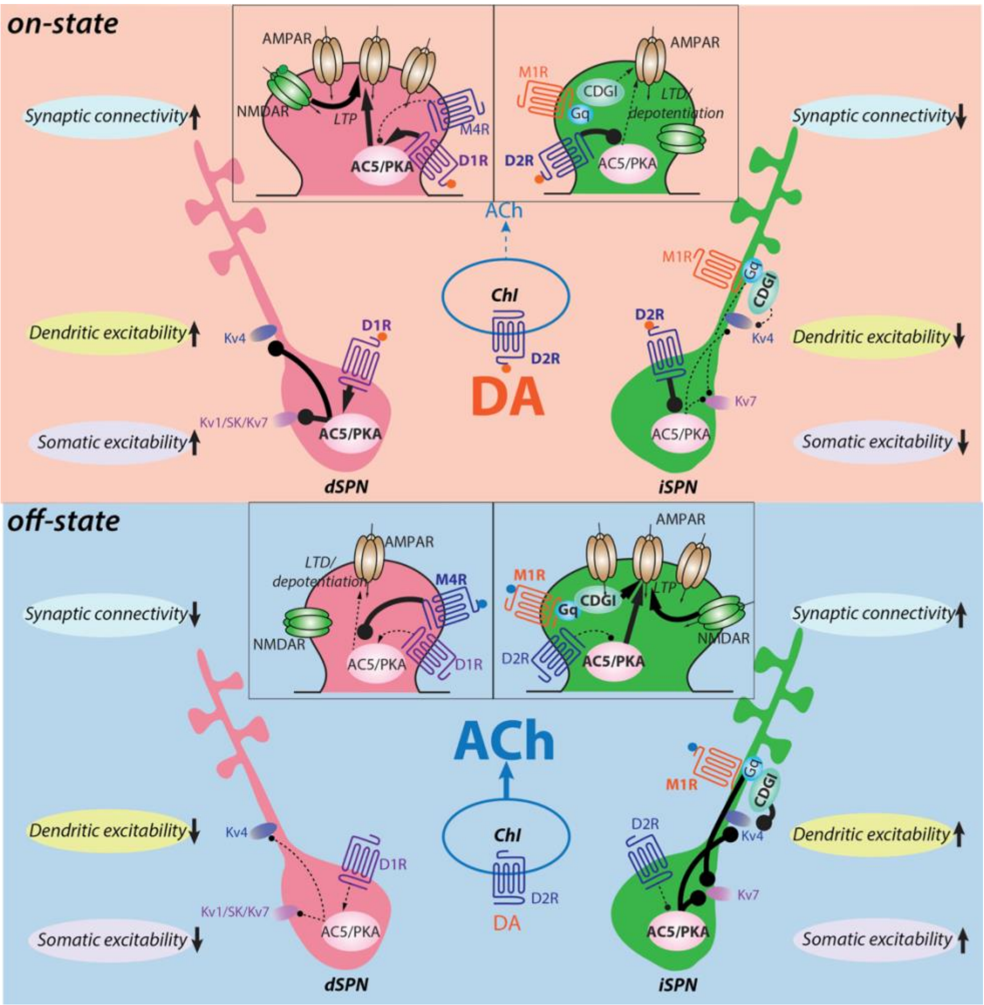

Fig. S4. Proposed signaling model of cell type-specific changes in SPNs in the on- and off-states of levodopa-induced dyskinesia (LID).

Insets, illustration of signaling pathways underlying state-dependent synaptic plasticity. Black arrowheads indicate positive regulation and black circles indicate negative regulation. AC5, adenylyl cyclase type 5; AMPAR, α-amino-3-hydroxyl-5-methyl-4-isoxazole-propionic acid receptor; Kv1, Kv1 voltage-dependent potassium channel (see ref. (*54*)); Kv4, Kv4 voltage-dependent potassium channel (see refs. (*23*, *54*)); Kv7, Kv7 voltage-dependent potassium channels (see ref. (*85*)); NMDAR, N-methyl-D-aspartate receptor PKA, protein kinase A; SK, small-conductance Ca^{2+}-activated potassium channel (see ref. (*54*)).

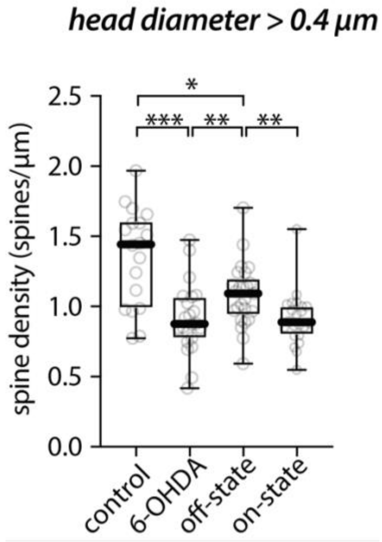

Fig. S5. Density of spines with larger head diameters detected by high-resolution confocal microscopy.

Box plot summary of the density of spines with > 0.4 µm head diameter in proximal dendrites of sparsely labeled iSPNs imaged by high-resolution confocal microscopy (control: n = 18 dendrites from 4 mice; 6-OHDA: n = 22 dendrites from 3 mice; off-state: n = 25 dendrites from 4 mice; on-state: n = 22 dendrites from 4 mice). This result was similar to the total spine density detected by 2PLSM (Fig. 4B). *** $p < 0.001$, ** $p < 0.01$, * $p < 0.05$, Mann-Whitney test.

Legends for Supplemental Videos:

Supplemental Video S1. Levodopa-induced dyskinetic behavior in unilaterally 6-OHDA-lesioned wildtype and CDGI KO mice. These videos of wildtype and CDGI KO mice were taken 40 min after intraperitoneal injection of levodopa (12mg/kg, supplemented with 12mg/kg benserazide) at the 5th AIM session. In contrast to the continuous dyskinesia in wildtype mouse, the dyskinesia exhibited by the CDGI KO mouse was intermittent, interruptible, and associated with more contralateral rotations.

Supplemental Video S2. Levodopa-induced dyskinetic behavior in unilaterally 6-OHDA-lesioned mice without or with M1R deletion from iSPNs. These videos were taken 40 min after levodopa administration at the 5th AIM session. Compared to the control mouse, the mouse with M1R deletion from iSPNs exhibited intermittent and reduced dyskinesia and more contralateral rotations.
